# Supplementary material for: A Computational Workflow to Predict Biological Target Mutations: The Spike Glycoprotein Case Study
Source: Molecules. 2023 Oct 14;28(20):7082. doi: 10.3390/molecules28207082 (PMC10609230; doi:10.3390/molecules28207082)
Supplement: Supplementary file 1 [file molecules-28-07082-s001.zip › molecules-2646881-supplementary.pdf]

Supplemental Materials

S1: Database structure

The database was designed as a standard relational SQL database. The table structure contains relevant information about collected sequences: id (internal name), name (name extracted from Gisaid), area (geographic area), date (date of collection), original (passage history of the encoded sequence), seqComplete (yes if there are no missing amino acid), ntd (N-terminal domain aminoacidic sequence), rbd (receptor binding domain aminoacidic sequence), ctd1 (C-terminal domain-1 aminoacidic sequence), ctd2 (C-terminal domain-2 aminoacidic sequence), s2 (central core aminoacidic sequence), mutsNtd, mutsRbd, mutsCtd1, mutsCtd2, mutsS2 register mutations in each domain of each sequence compared to the Wuhan sequence.

seqsCov19HumanOriginal	
**id**	**varchar**
name	varchar
area	varchar
dateYYYY_MM_DD	date
original	varchar
seqComplete	varchar
ntd	varchar
rbd	varchar
ctd1	varchar
ctd2	varchar
s2	varchar
mutsNtd	varchar
mutsRbd	varchar
mutsCtd1	varchar
mutsCtd2	varchar
mutsS2	varchar

Figure S1: SQL table format

S2Analysis of the effect of known mutations

A. The Intermolecular HINT score of representative RBD-antibodies complexes.

	HINT Intermolecular Score					
Ref. PDB	WT	Alpha	Beta	Gamma	Delta	BA.1
6XC4	2.85E+03	2.91E+03	2.56E+03	2.56E+03	2.57E+03	2.07E+03
7KN4	2.43E+03	2.72E+03	2.11E+03	2.04E+03	2.14E+03	2.44E+03
7LOP	1.66E+03	1.65E+03	1.08E+03	1.01E+03	1.60E+03	- 1.42E+03
6YZ5	2.10E+03	2.02E+03	2.52E+02	2.92E+02	1.94E+03	5.25E+02
7BWJ	9.05E+02	8.83E+02	1.78E+02	1.92E+02	5.59E+02	2.77E+02
7EAN	3.92E+03	3.94E+03	3.89E+03	3.86E+03	3.81E+03	3.98E+03
7M7W	6.01E+03	5.86E+03	5.87E+03	5.89E+03	5.88E+03	5.01E+03
7DPM	1.56E+03	1.77E+03	1.74E+03	1.72E+03	2.03E+03	1.28E+03
7JX3	8.84E+02	1.01E+03	1.18E+03	1.00E+03	9.77E+02	9.95E+02
7M3I	1.91E+03	2.11E+03	2.11E+03	2.16E+03	1.96E+03	2.18E+03

Table S2.A. Alpha, beta, gamma, delta and BA.1 mutations were modeled on the wild-type structure. Alpha complexes present a comparable intermolecular score than the wildtype. Other variants, characterized by more than a single point mutation, present a significant reduction of affinity (red).

B. The Intermolecular HINT score of RBD in complex with known monoclonal antibodies

		HINT Intermolecular Score					
mAb	PDB ID	WT	Alpha	Beta	Gamma	Delta	BA.1
LYCOV-555	7KMG	2.13E+03	2.12E+03	8.71E+02	7.48E+02	1.72E+03	6.68E+02
LYCOV016	7C01	3.26E+03	3.29E+03	2.41E+03	2.28E+03	3.29E+03	2.04E+03
REGN10933	6XDG	2.13E+03	2.17E+03	1.46E+03	7.49E+02	1.97E+03	8.71E+02
REGN10987	6XDG	1.53E+03	1.40E+03	7.63E+02	6.42E+02	9.98E+02	3.24E+02
S309	7R6X	1.35E+03	1.04E+03	1.35E+03	1.29E+03	1.60E+03	1.21E+03
COV2-2130	7L7E	2.50E+03	2.50E+03	1.56E+03	1.61E+03	2.51E+03	9.82E+02
COV2-2196	7L7E	2.26E+03	2.23E+03	1.67E+03	2.05E+03	1.55E+03	1.44E+03
S304	7R6X	8.84E+02	1.01E+03	1.18E+03	1.00E+03	9.77E+02	9.95E+02

TableS2. B. *Alpha, beta, gamma, delta and BA.1 mutations were modeled on the wild-type structure. Alpha complexes present a comparable intermolecular score than the wildtype. Other variants, characterized by more than a single point mutation, present a significant reduction of affinity (red). BA.1 variant presents a significant affinity reduction toward all the analyzed complexes for the exception of S309 (sotrovimab) and S304*

S3: List of the 55 mutable residues

K444, Y449, N450, L452, L455, S459, N460, P463, T470, E471, Q474, A475, G476, S477, T478, P479, E484, F490, Q493, S494, Q498, P499, N501, Y505, R457, Y473, N487, T500, Y489, G339, E340, A344,

R346, A348, A352, D405, E406, R408, Q414, T415, A419, K417, D420, N370, S371, S373, S375, S376, D427, D428, E516, H519, A520, P521, A522.

Mutable residues were divided into three different groups: A (blue residues), B (red residues) and C (green residues) such that the residues of one were 10 Å away from those of the others. Most of the residues of group A are in the main random coil region responsible for the interaction with the target

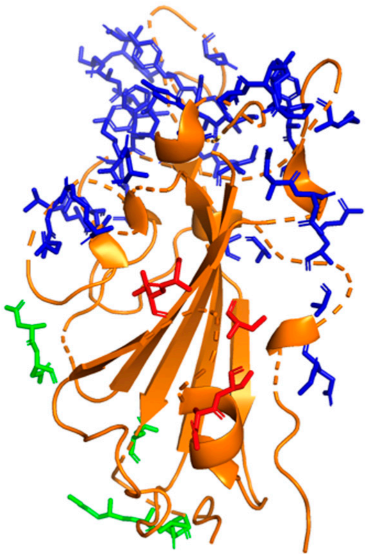

Group A: K444, Y449, N450, L452, L455, S459, N460, P463, T470, E471, Q474, A475, G476, S477, T478, P479, E484, F490, Q493, S494, Q498, P499, N501, Y505, R457, Y473, N487, T500, Y489, G339, E340, A344, R346, A348, A352, D405, E406, R408, Q414, T415, A419, K417, D420

Group B: N370, S371, S373, S375, T376

Group C: D427, D428, E516, H519, A520, P521, A522

S4: Intramolecular stability of generated models

All the generated models were analyzed in HINT, using the Intramolecular option, and compared to the reference system.

Reference system is characterized by mutations shared between BA.1 and BA.2 variants.

This analysis allows the identification of mutants less stable (red), stable as (yellow) and more stable than the refence system.

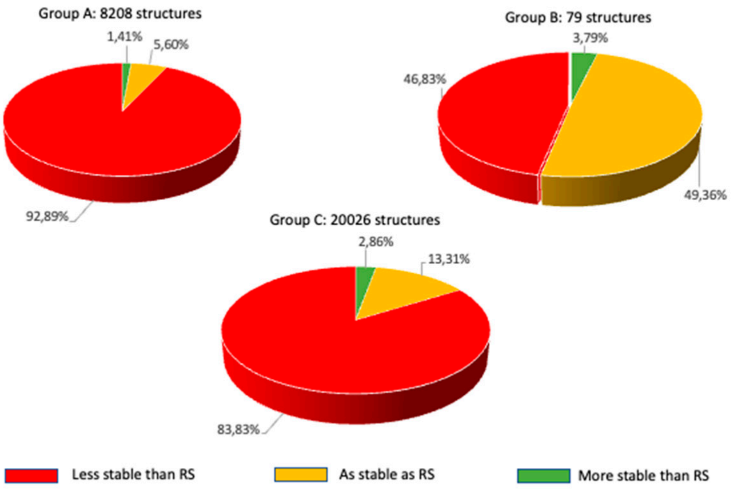

S5. Analysis of most stable generated models

The most stable RBD mutated proteins, belonging to group A and predicted with our method, were deepen analyzed in order to evaluate the affinity with the target and the stability of the resulted complexes. The intermolecular and the intramolecular HINT scoring function were used.

Moreover, a molecular dynamics simulation of all these systems was carried out in order to evaluate their stability during the simulation time. The average number of hydrogen bonds between chainA (ACE2) and chainB (RBD) was calculated.

Internal Number	Mutations	RBD Stability	RBD-ACE2 Affinity	RBD-ACE2 Stability	RMSD (average)	Average number of h-bonds
**1muts6**	A419S	1.17E+04	1.52E+03	1.12E+05	0.93	16
**1muts9**	A475S	1.19E+04	1.57E+03	1.12E+05	0.90	12
**1muts31**	A344S	1.18E+04	1.47E+03	1.12E+05	0.65	18
**1muts32**	A344D	1.15E+04	1.44E+03	1.11E+05	0.85	11
**1muts37**	A348S	1.21E+04	1.37E+03	1.12E+05	0.95	15
**1muts39**	A348E	1.17E+04	1.53E+03	1.13E+05	0.72	12
**1muts64**	Q414K	1.17E+04	1.59E+03	1.12E+05	0.59	11
**1muts88**	L452R	1.16E+04	1.42E+03	1.12E+05	1.12	12
**2muts1**	Q414R, A419T	1.19E+04	1.04E+03	1.11E+05	0.81	14
**2muts4**	A419T, G476D	1.23E+04	1.11E+03	1.12E+05	0.89	13
**2muts19**	A419T, A475D	1.17E+04	1.09E+03	1.11E+05	0.86	13
**2muts45**	A348E, A419T	1.20E+04	1.49E+03	1.12E+05	1.08	13

**2muts196**	A419S, E471A	1.16E+04	1.55E+03	1.13E+05	0.91	11
**2muts257**	P463T, T500I	1.15E+04	1.24E+03	1.12E+05	0.99	12
**2muts305**	A419S, P463R	1.19E+04	1.65E+03	1.12E+05	0.97	16
**2muts337**	A419S, T470A	1.20E+04	1.53E+03	1.12E+05	0.91	13
**2muts1006**	K444M, A419S	1.22E+04	1.42E+03	1.12E+05	1.10	11
**2muts1013**	A419S, K444T	1.17E+04	1.45E+03	1.13E+05	0.73	13
**2muts1149**	A419S, D420G	1.17E+04	1.41E+03	1.13E+05	0.87	11
**2muts1151**	A419S, D420H	1.18E+04	1.51E+03	1.13E+05	0.91	14
**2muts1153**	A419S, D420N	1.19E+04	1.43E+03	1.11E+05	0.85	16
**2muts1424**	A419S, Y449F	1.18E+04	1.13E+03	1.12E+05	0.98	12
**2muts1522**	A419S, L452R	1.22E+04	1.41E+03	1.12E+05	1.03	17
**2muts2539**	R408S, A419S	1.18E+04	1.52E+03	1.12E+05	0.99	18
**2muts3232**	A352D, T500P	1.16E+04	1.23E+03	1.11E+05	0.81	16
**2muts3298**	A352T, P499R	1.16E+04	1.25E+03	1.12E+05	1.08	14
**2muts3302**	A352D, P499R	1.16E+04	1.20E+03	1.12E+05	0.67	16
**2muts3323**	A352D, A419S	1.22E+04	1.47E+03	1.12E+05	0.91	13
**2muts3410**	A348E, A352T	1.19E+04	1.54E+03	1.11E+05	0.87	15
**2muts3412**	A348T, S494A	1.15E+04	1.45E+03	1.11E+05	1.05	16
**2muts3426**	A348T, D405Y	1.16E+04	1.35E+03	1.10E+05	1.09	16
**2muts3464**	A348E, E406Q	1.16E+04	1.48E+03	1.11E+05	1.01	16
**2muts3494**	A348E, N460H	1.17E+04	1.52E+03	1.11E+05	0.91	12
**2muts3496**	A348E, N460K	1.16E+04	1.49E+03	1.12E+05	0.99	13
**2muts3500**	A348S, A419S	1.30E+04	1.58E+03	1.13E+05	0.97	13
**2muts3502**	A348E, A419S	1.22E+04	1.53E+03	1.12E+05	0.86	15

**2muts3523**	A348T, P463H	1.18E+04	1.61E+03	1.12E+05	0.94	15
**2muts3524**	A348E, P463S	1.18E+04	1.41E+03	1.11E+05	0.92	16
**2muts3538**	A348S, E471Q	1.16E+04	1.41E+03	1.12E+05	1.08	16
**2muts3582**	A348E, T500I	1.15E+04	1.20E+03	1.11E+05	0.97	15
**2muts3659**	E340G, A348E	1.16E+04	1.40E+03	1.12E+05	0.96	17
**2muts4445**	A344S, R346S	1.16E+04	1.59E+03	1.13E+05	1.10	12
**2muts4564**	A344D, T470A	1.20E+04	1.52E+03	1.12E+05	0.88	12
**2muts4584**	A344D, Y473H	1.15E+04	1.55E+03	1.13E+05	1.07	11
**2muts4617**	A344T, N460H	1.15E+04	1.59E+03	1.12E+05	1.02	9
**2muts4627**	A344D, A419S	1.27E+04	1.58E+03	1.13E+05	0.84	15
**2muts4667**	A344D, Y449F	1.16E+04	1.26E+03	1.11E+05	1.02	13
**2muts4697**	A344S, A352D	1.16E+04	1.41E+03	1.12E+05	0.91	12
**2muts4698**	A344S, A352S	1.18E+04	1.48E+03	1.11E+05	0.93	15
**2muts4699**	A344S, A352T	1.16E+04	1.50E+03	1.13E+05	0.97	16
**2muts4728**	A344D, S494L	1.16E+04	1.50E+03	1.11E+05	0.80	18
**2muts5460**	A344S, P479T	1.16E+04	1.37E+03	1.11E+05	1.05	13
**2muts5602**	A419S, P479H	1.19E+04	1.54E+03	1.11E+05	0.53	15
**2muts5837**	A348S, P479S	1.20E+04	1.47E+03	1.12E+05	0.65	13
**2muts5880**	A348E, Q414P	1.17E+04	1.27E+03	1.11E+05	1.04	12
**2muts5887**	A348T, Q414K	1.20E+04	1.40E+03	1.11E+05	0.43	16
**2muts6169**	Q414R, Y473F	1.17E+04	1.32E+03	1.11E+05	0.82	17
**2muts6191**	Q414K, P463H	1.17E+04	1.57E+03	1.12E+05	1.00	18
**2muts6253**	Q414R, N460K	1.16E+04	1.45E+03	1.11E+05	0.94	15
**2muts6256**	Q414P, A419S	1.16E+04	1.58E+03	1.12E+05	0.83	17

**2muts6321**	E406K, Q414R	1.15E+04	1.82E+03	1.11E+05	0.82	16
**2muts6355**	Q414R, S459P	1.19E+04	1.50E+03	1.11E+05	0.86	16
**2muts6536**	Q414R, Q474H	1.16E+04	1.45E+03	1.11E+05	1.05	15
**2muts6574**	A344S, Q474H	1.16E+04	1.45E+03	1.12E+05	1.00	15
**2muts6776**	A419S, Q474H	1.20E+04	1.55E+03	1.12E+05	1.01	14
**2muts6853**	A463H, Q474H	1.15E+04	1.45E+03	1.11E+05	0.85	13
**2muts7162**	A348S, Q474P	1.18E+04	1.55E+03	1.12E+05	0.93	11
**2muts7235**	A348D, G476C	1.18E+04	9.48E+02	1.11E+05	0.59	12
**2muts7246**	T415P, G476D	1.17E+04	1.30E+03	1.11E+05	0.68	12
**2muts7256**	G476C, N487D	1.21E+04	1.35E+03	1.11E+05	0.68	16
**2muts7358**	L452Q, G476D	1.15E+04	1.21E+03	1.11E+05	0.92	14
**2muts7452**	P463S, G476D	1.19E+04	1.31E+03	1.11E+05	0.86	13
**2muts7512**	A419S, G476D	1.18E+04	1.53E+03	1.11E+05	0.99	15
**2muts7619**	A344T, G476C	1.17E+04	1.19E+03	1.11E+05	1.04	17
**2muts7671**	Q414K, G476D	1.21E+04	1.35E+03	1.12E+05	0.81	15
**2muts7672**	Q414L, G476D	1.16E+04	1.36E+03	1.11E+05	0.96	16
**2muts7683**	A475S, G476C	1.17E+04	1.25E+03	1.11E+05	0.96	17
**2muts7688**	Q414R, A475T	1.17E+04	1.62E+03	1.12E+05	0.99	13
**2muts7728**	A344S, A475S	1.22E+04	1.53E+03	1.12E+05	0.81	12
**2muts7732**	A344D, A475T	1.21E+04	1.61E+03	1.12E+05	1.05	11
**2muts7736**	E340V, A475S	1.16E+04	1.59E+03	1.12E+05	1.02	11
**2muts7766**	A352D, A475S	1.18E+04	1.54E+03	1.12E+05	0.81	15
**2muts7850**	A475S, P499S	1.15E+04	1.25E+03	1.11E+05	1.07	17
**2muts7889**	T470P, A475D	1.15E+04	1.20E+03	1.12E+05	0.85	14

**2muts7941**	L452R, A475S	1.17E+04	1.36E+03	1.12E+05	0.81	14	
**2muts7999**	A475T, N487D	1.19E+04	1.57E+03	1.12E+05	0.73	13	
**2muts8020**	A348T, A475T	1.18E+04	1.69E+03	1.11E+05	0.83	11	
**2muts8047**	A475S, P479S	1.17E+04	1.55E+03	1.12E+05	0.98	16	
**2muts8052**	L455S, A475T	1.16E+04	2.65E+03	1.13E+05	0.96	17	
**2muts8053**	L455S, A475S	1.16E+04	2.54E+03	1.13E+05	0.95	18	
**2muts8064**	A348E, L455S	1.17E+04	2.32E+03	1.12E+05	0.92	15	
**2muts8066**	A348T, L455S	1.15E+04	2.54E+03	1.14E+05	1.05	16	
**2muts8098**	L455S, T500P	1.17E+04	2.34E+03	1.13E+05	0.80	18	
**2muts8176**	L455S, G476S	1.15E+04	2.36E+03	1.13E+05	1.02	15	

S6: RMSF calculation

The root mean square fluctuation (RMSF) over trajectory was calculated for all systems.

X axis: Residue number

Y axis: RMSF (nm)

<table>
<tr><th>Model</th><th>RMSF (RDB)</th><th>RMSF (ACE2)</th></tr>
<tr><td>WT</td><td>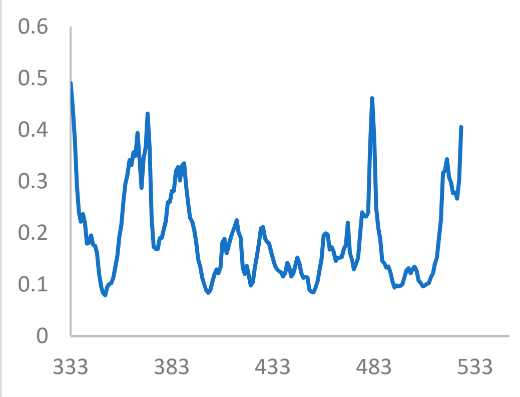
</td><td>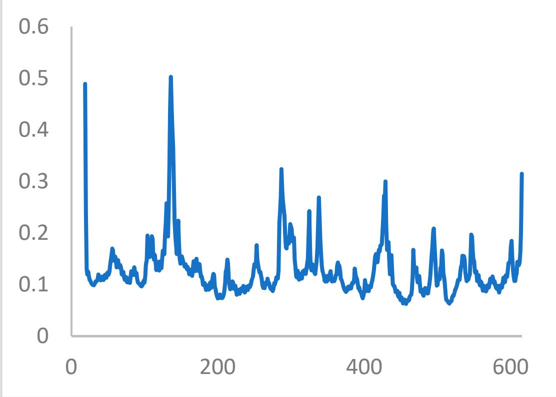
</td></tr>
</table>

Alpha

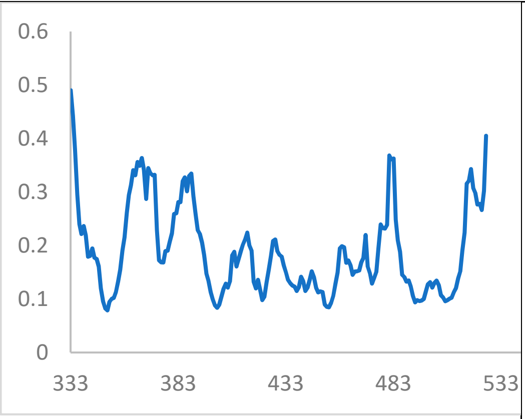

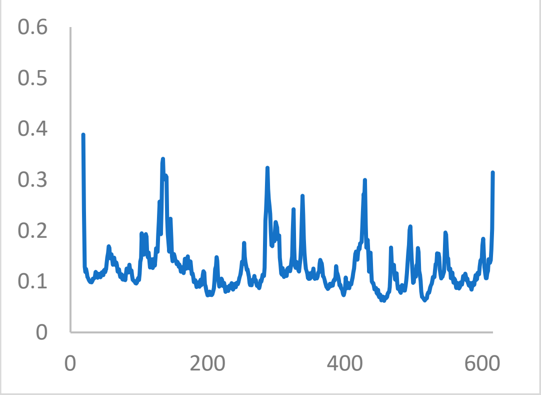

Beta

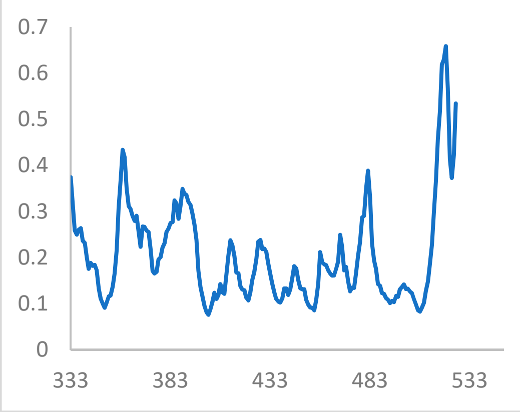

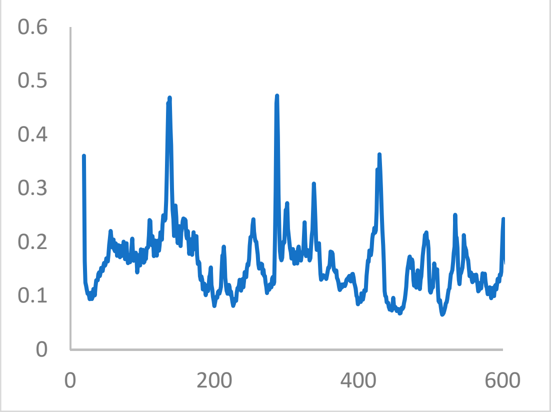

Delta

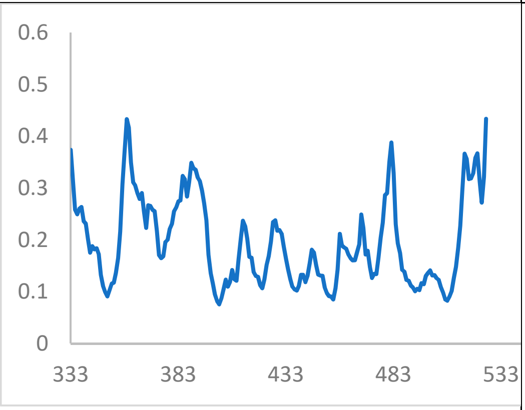

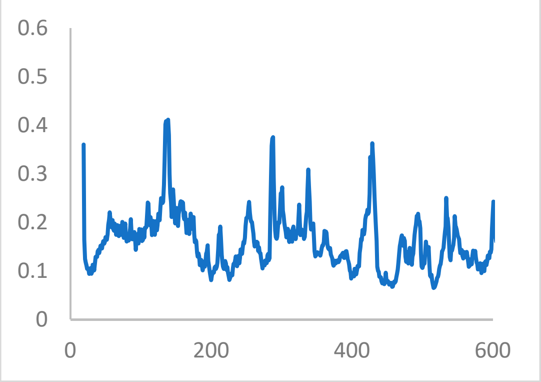

Omicron

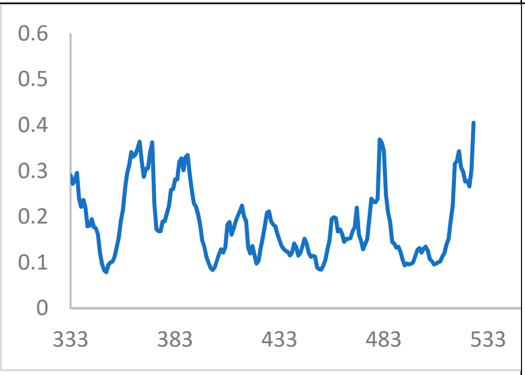

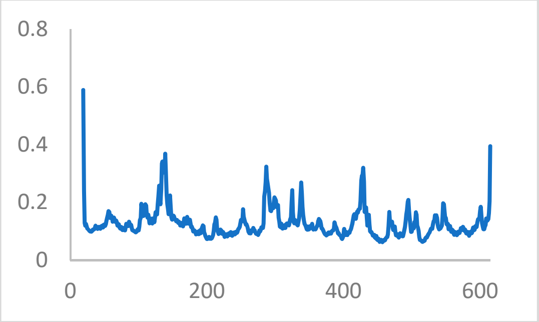

1muts6	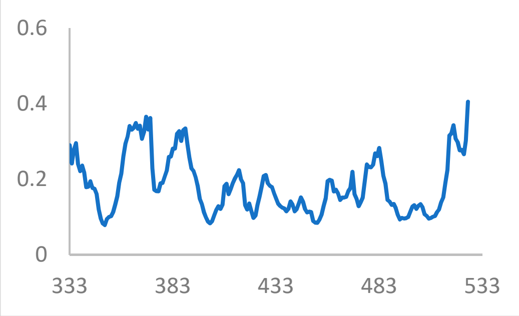	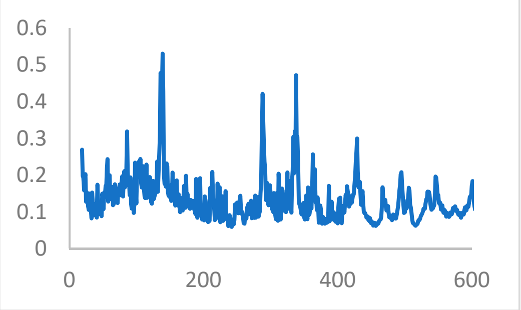
1muts9	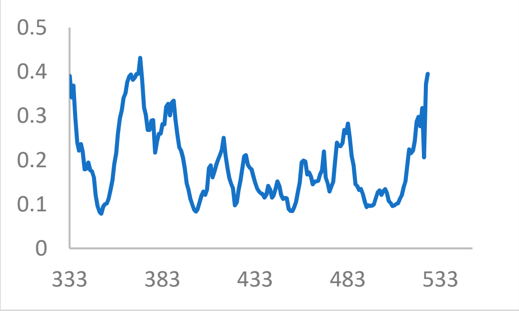	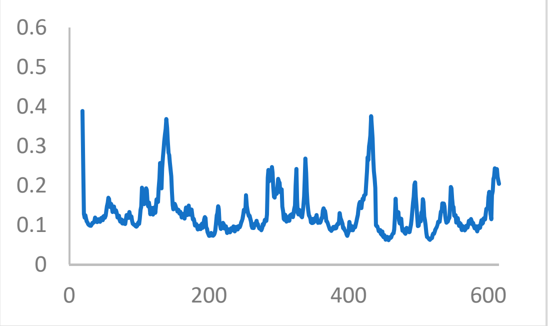
1muts31	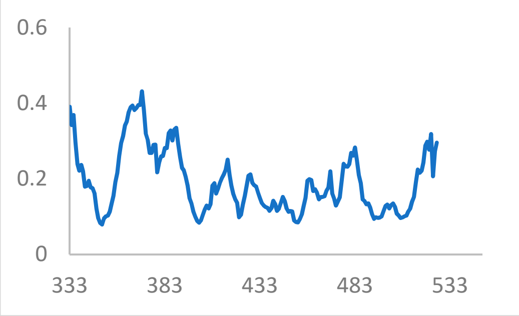	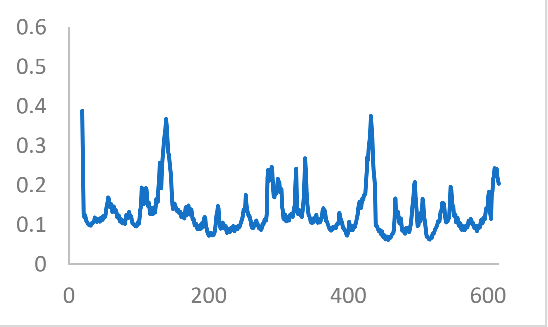
1muts32	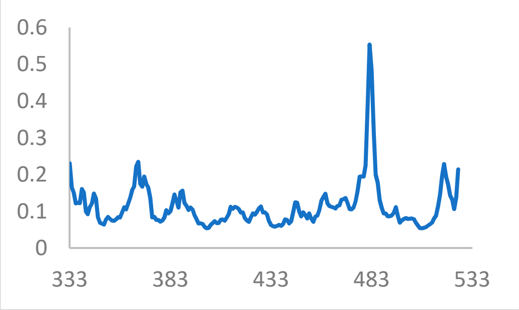	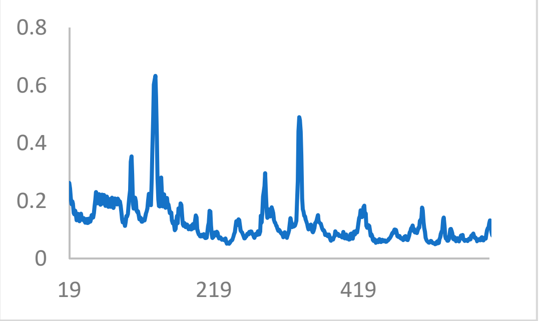
1muts37	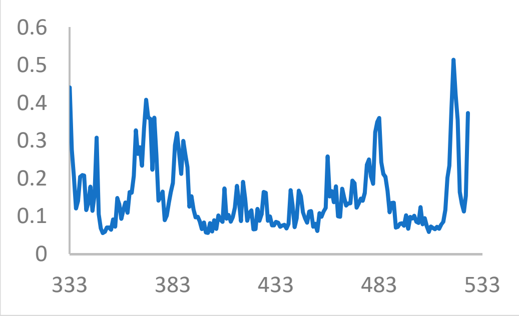	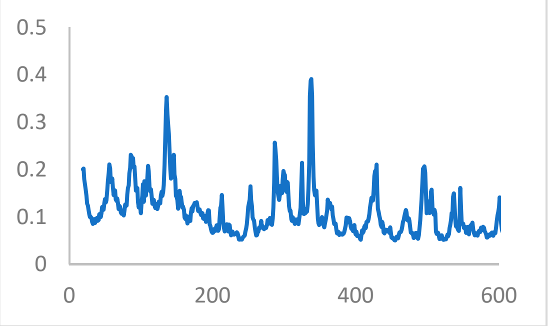

1muts39

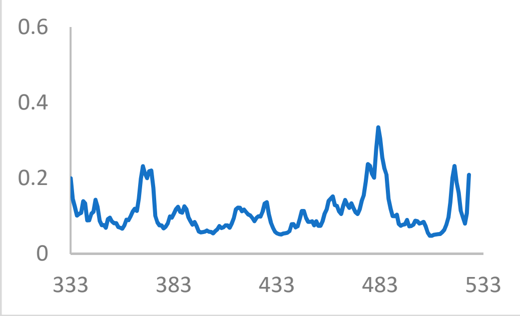

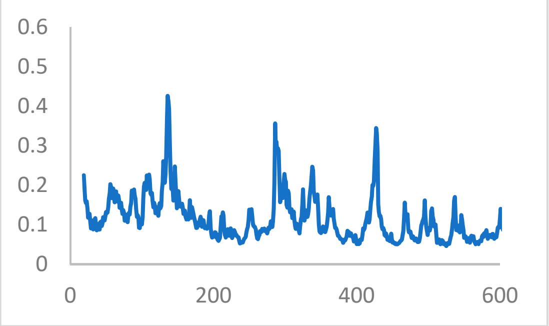

1muts64

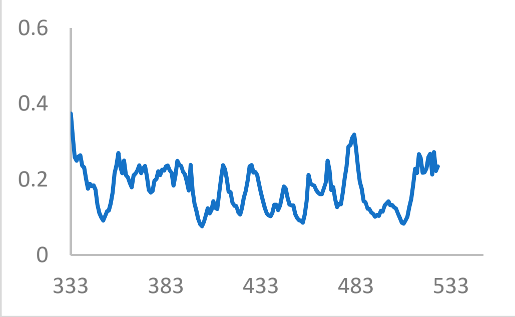

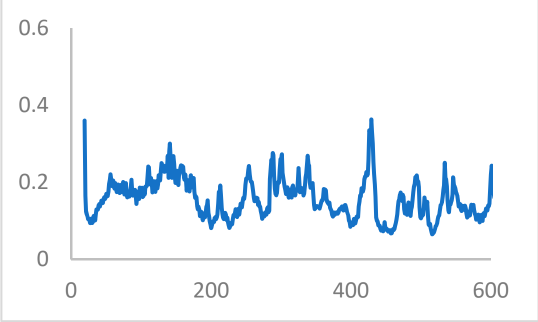

1muts88

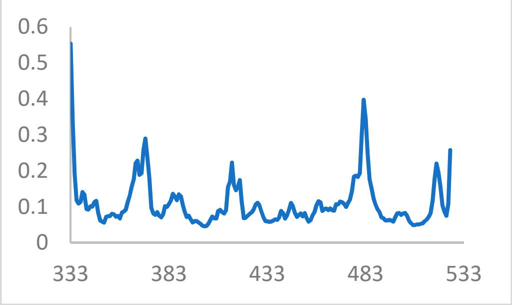

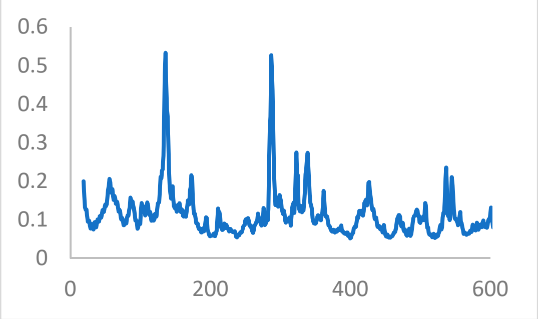

2muts1

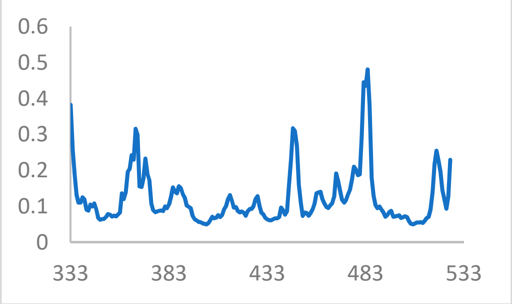

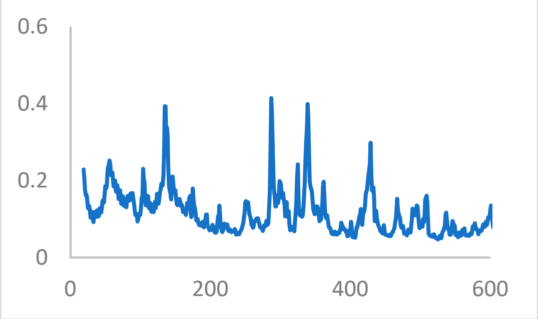

2muts4

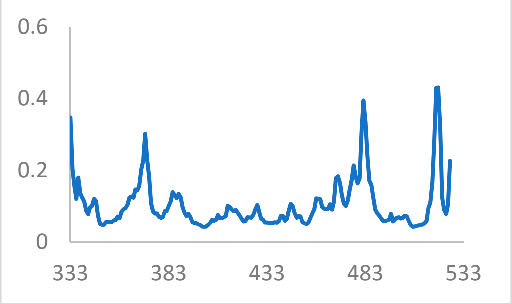

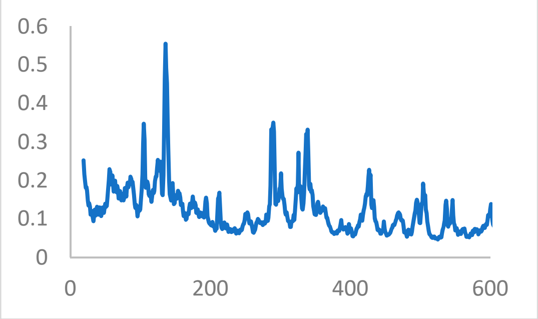

<table>
<tr><td>2muts19</td><td>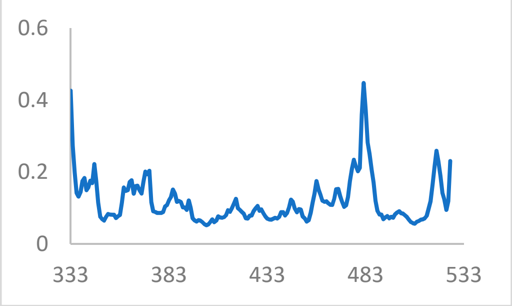
</td><td>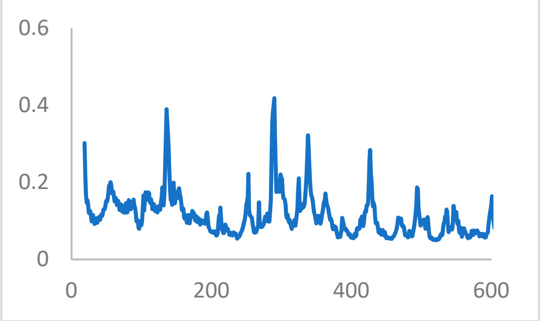
</td></tr>
<tr><td>2muts45</td><td>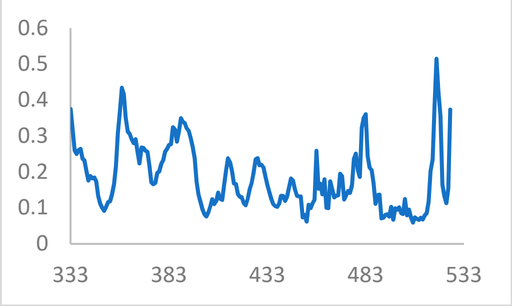
</td><td>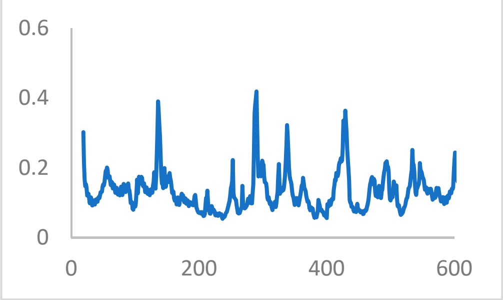
</td></tr>
<tr><td>2muts196</td><td>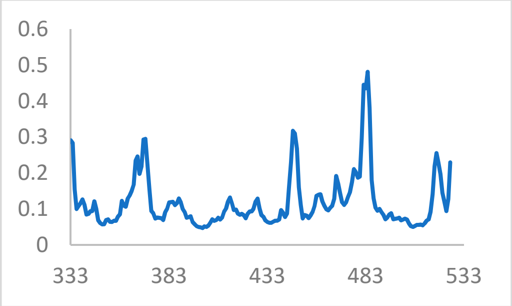
</td><td>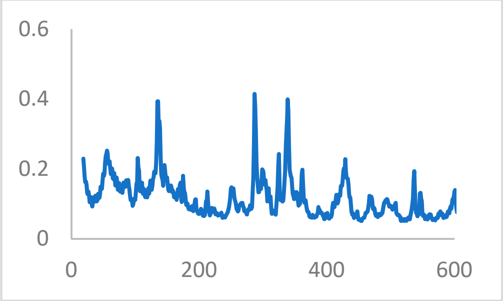
</td></tr>
<tr><td>2muts257</td><td>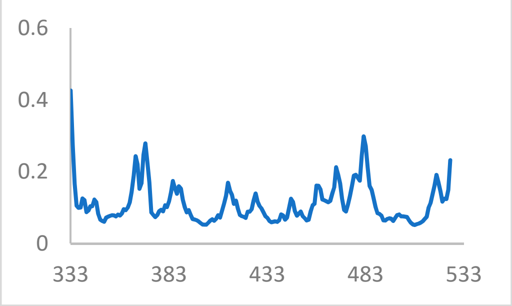
</td><td>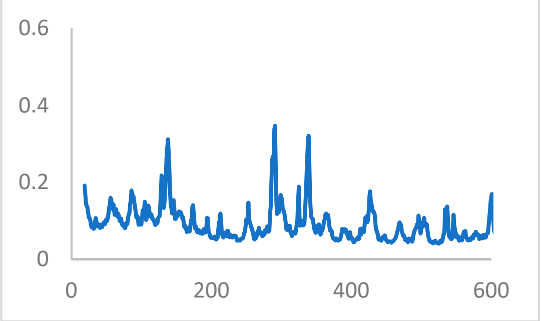
</td></tr>
<tr><td>2muts305</td><td>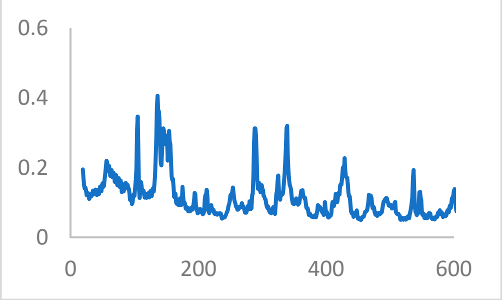
</td><td>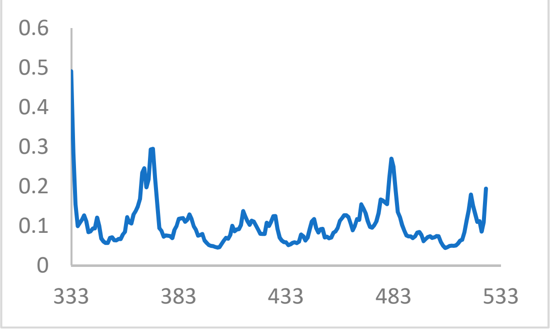
</td></tr>
</table>

2muts337

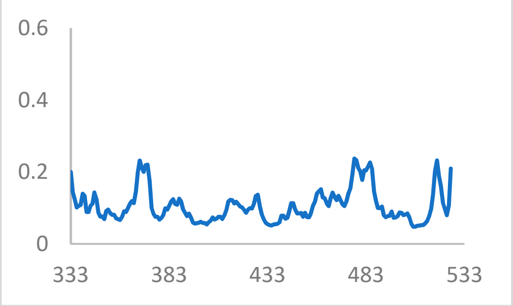

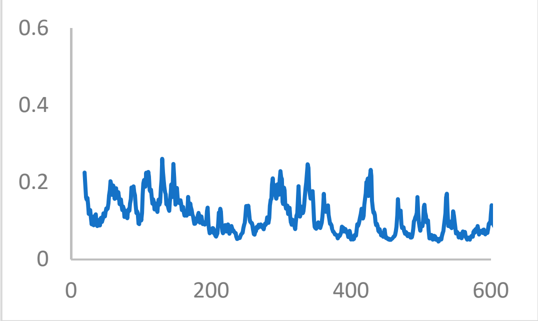

2muts1006

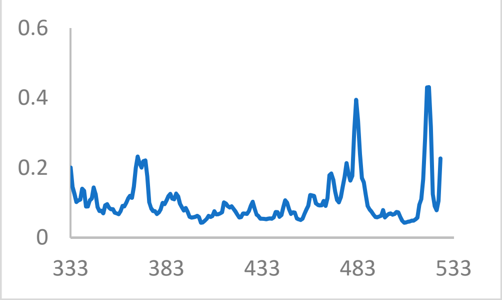

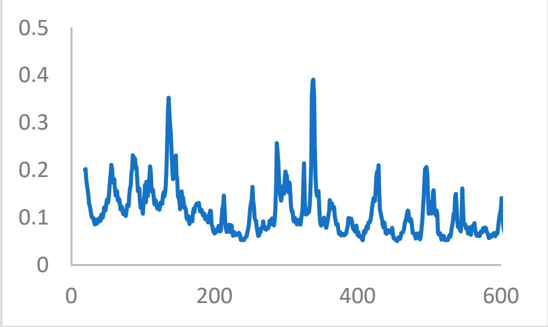

2muts1013

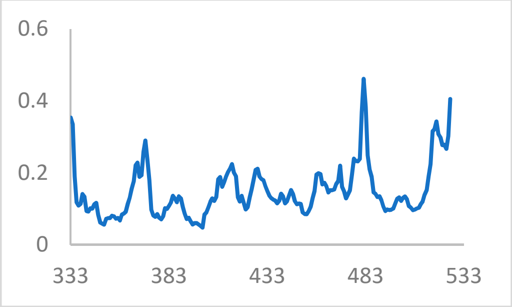

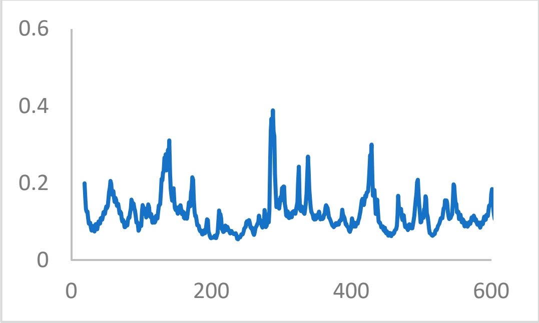

2muts1149

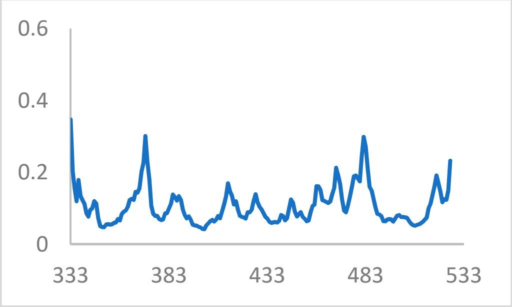

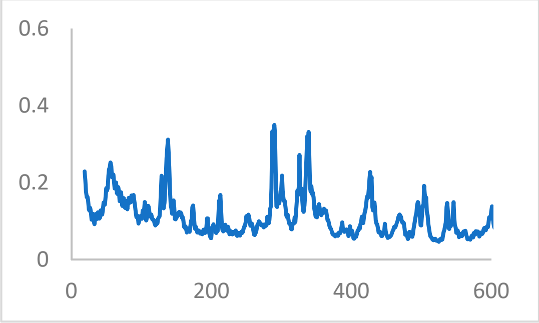

2muts1151

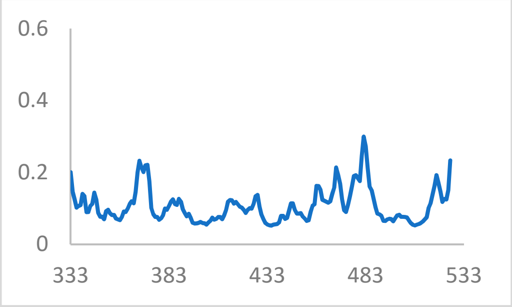

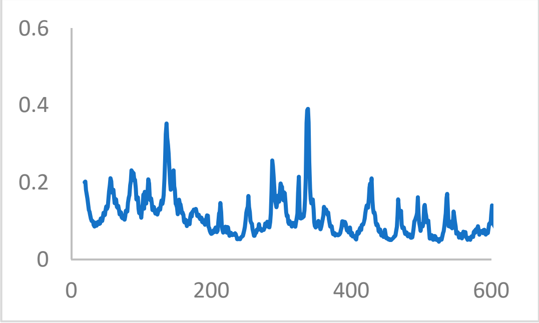

<table>
<tr><td>2muts1153</td><td>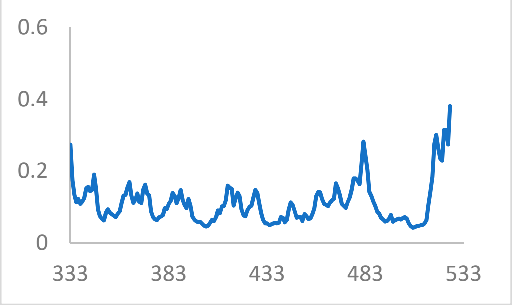</td><td>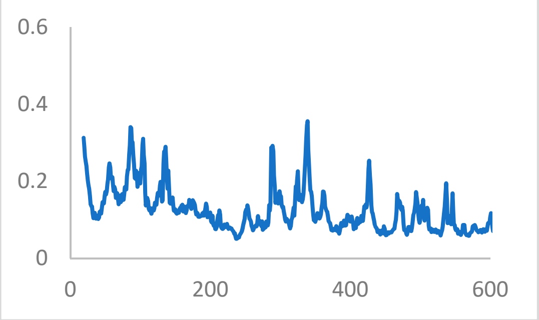</td></tr>
<tr><td>2muts1424</td><td>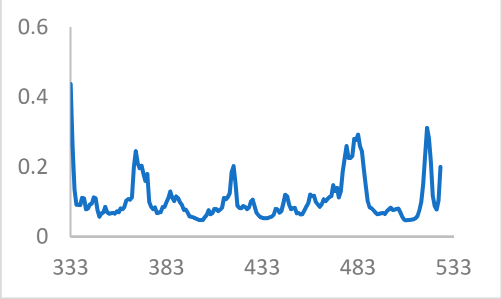</td><td>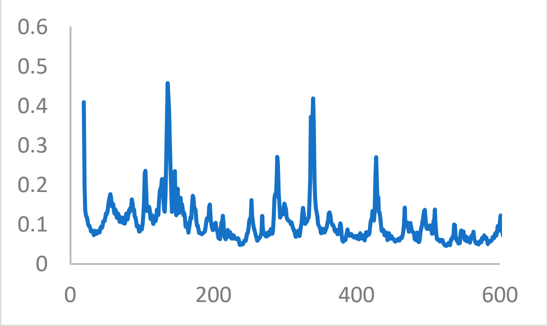</td></tr>
<tr><td>2muts1522</td><td>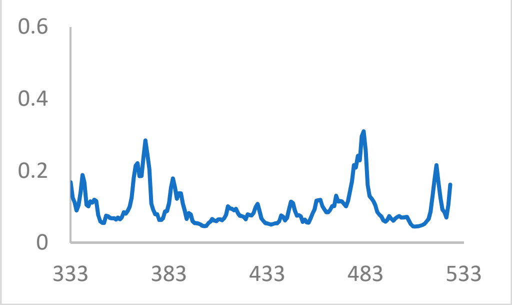</td><td>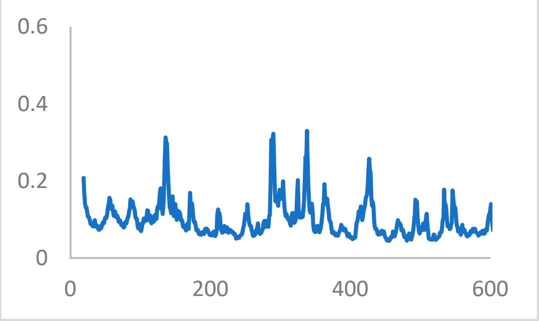</td></tr>
<tr><td>2muts2539</td><td>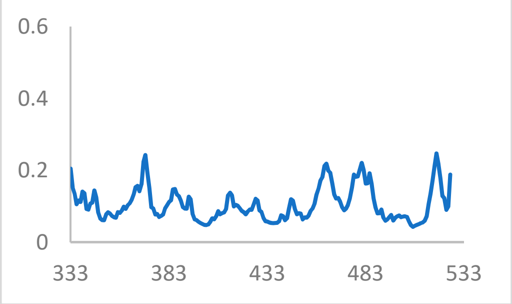</td><td>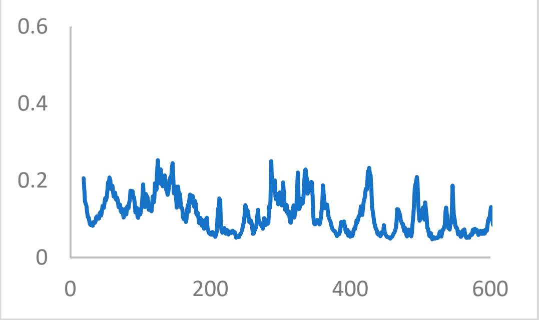</td></tr>
<tr><td>2muts3232</td><td>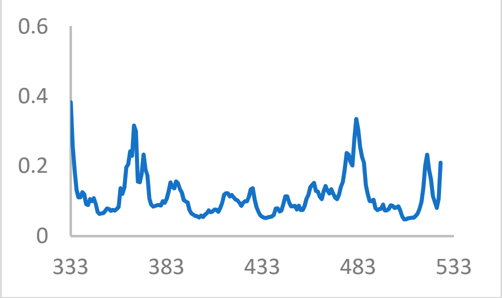</td><td>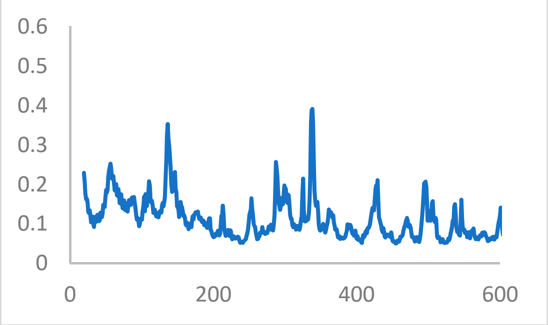</td></tr>
</table>

2muts3298	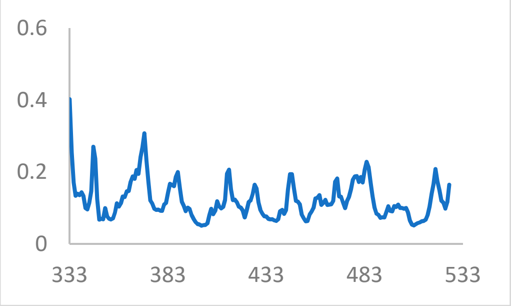	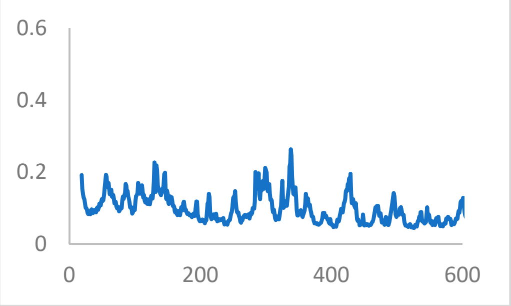
2muts3302	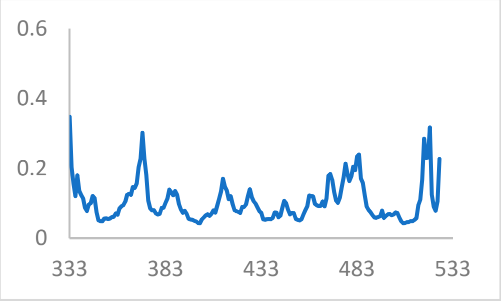	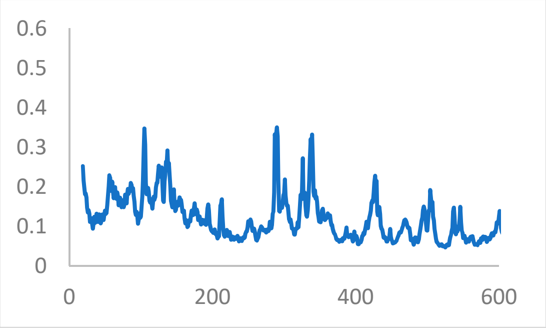
2muts3323	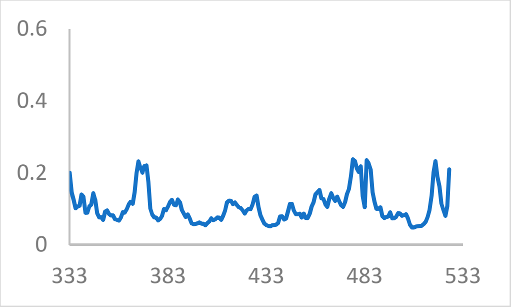	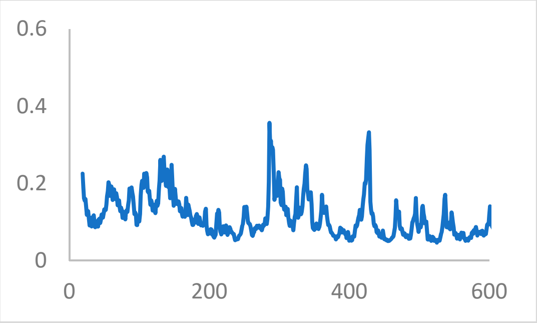
2muts3410	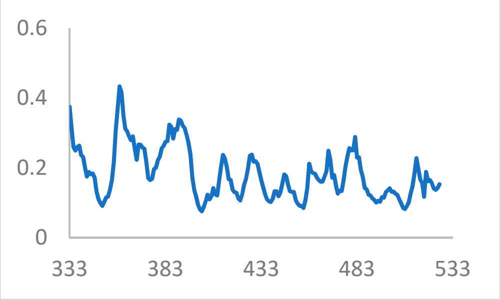	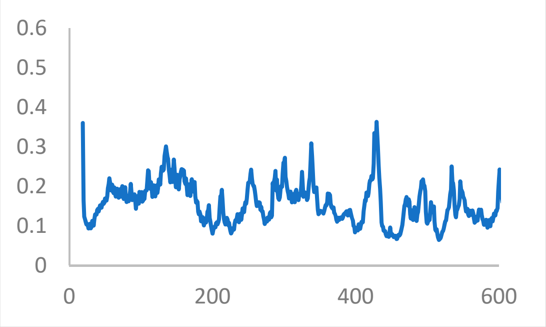
2muts3412	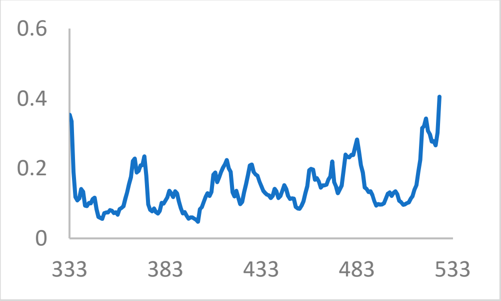	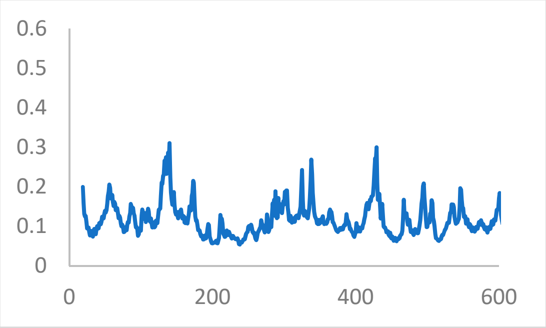

2muts3426	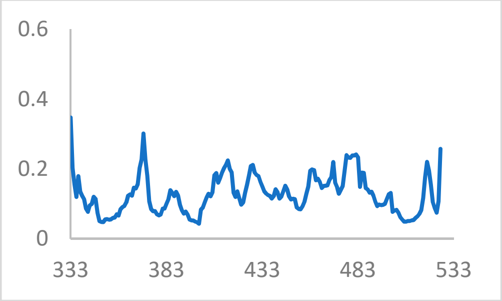	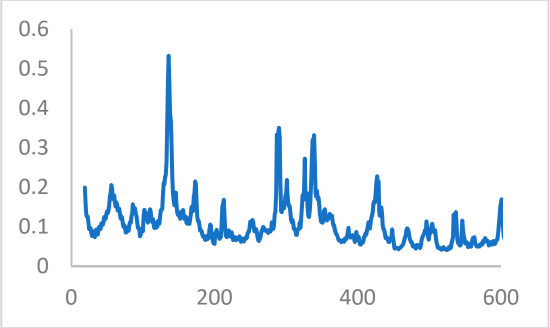
2muts3464	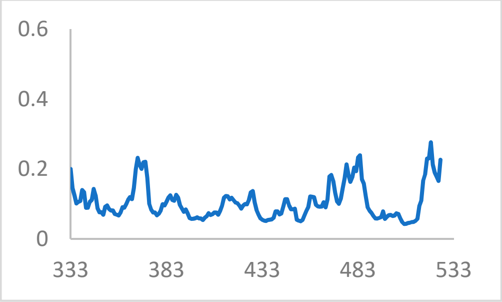	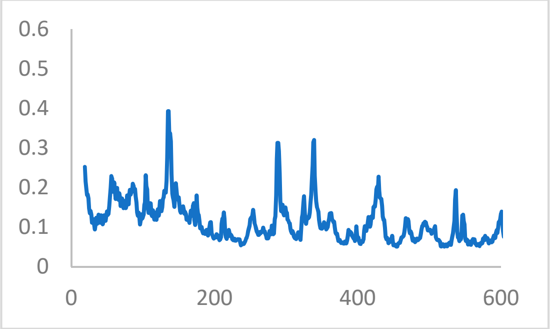
2muts3494	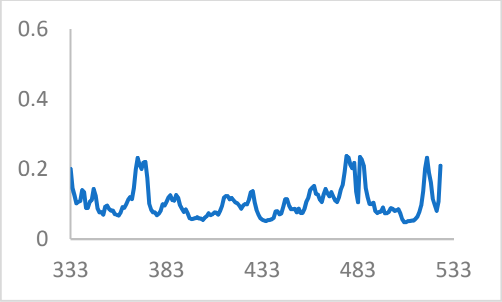	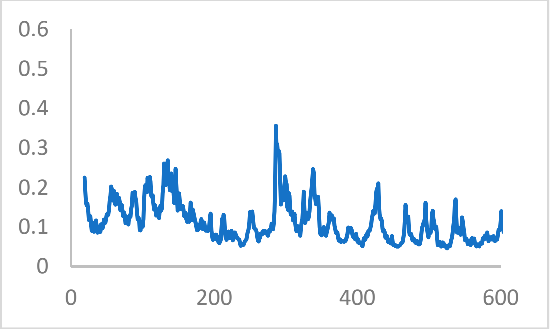
2muts3496	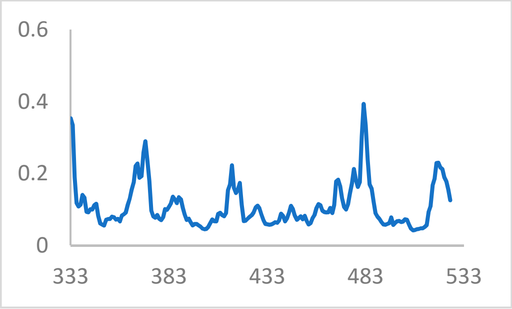	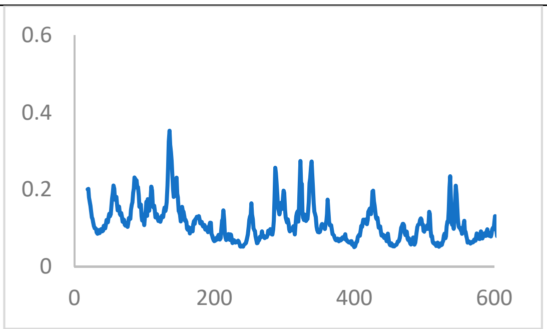
2muts3500	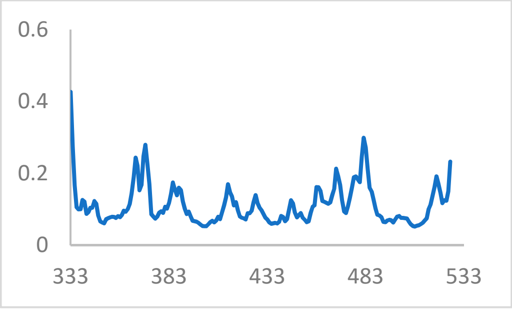	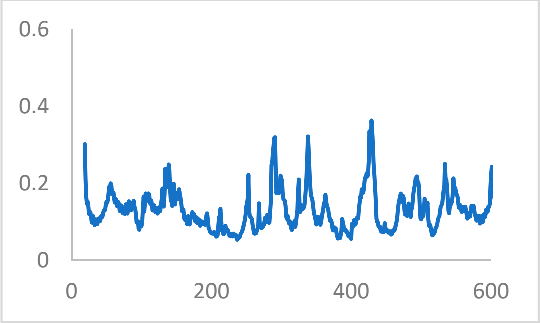

2muts3502	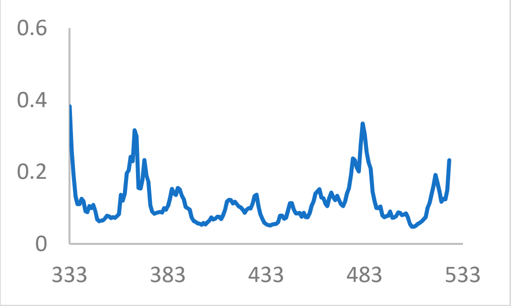	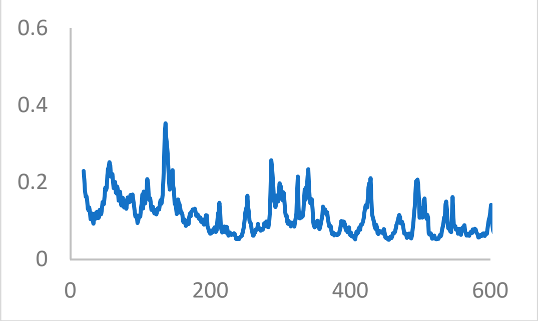
2muts3523	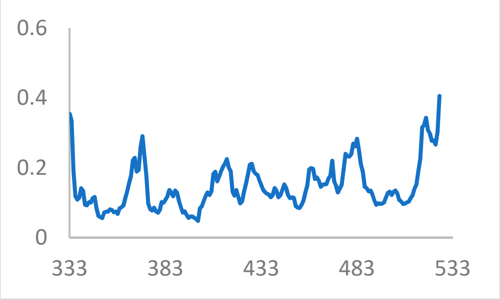	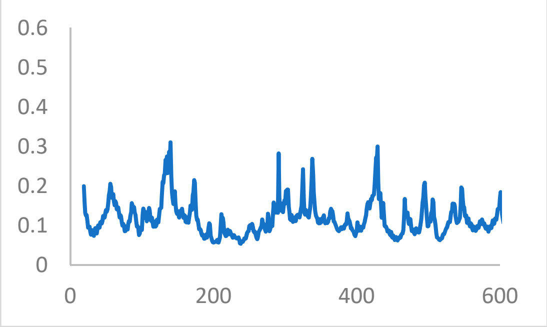
2muts3524	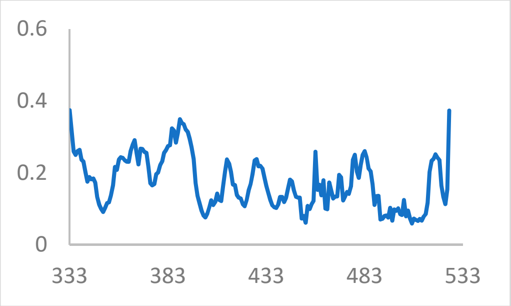	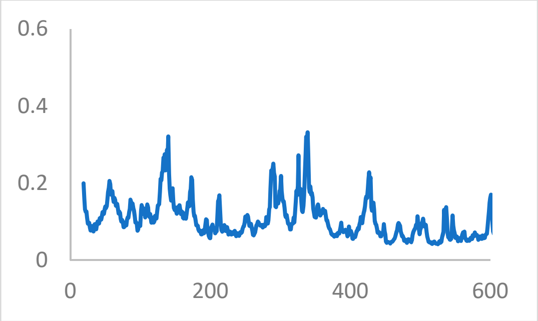
2muts3538	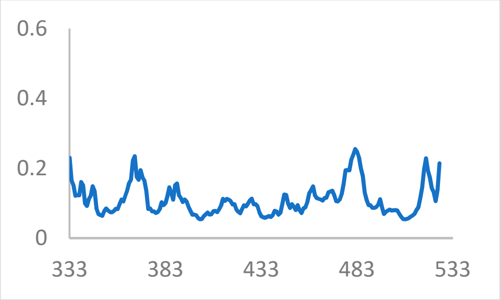	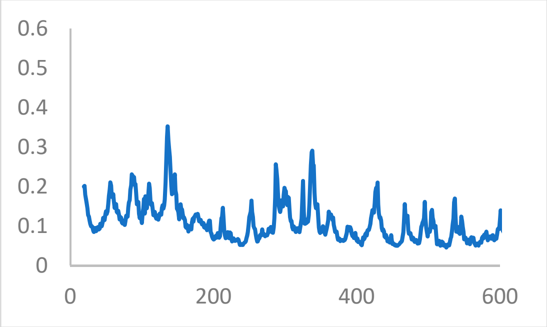
2muts3582	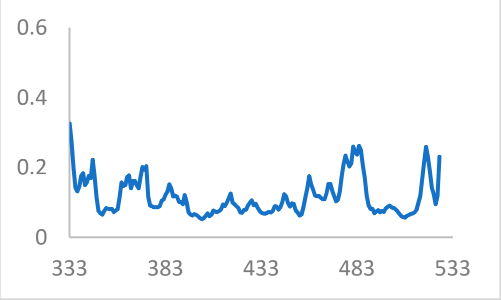	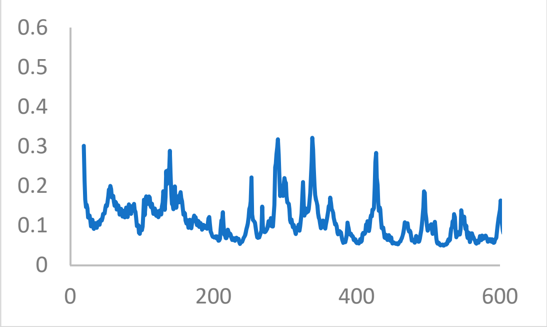

2muts3659

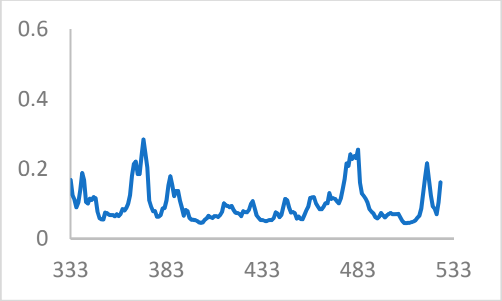

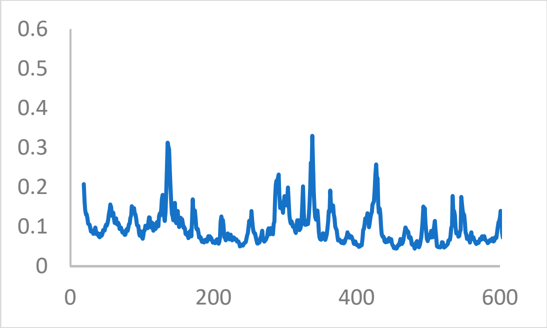

2muts4445

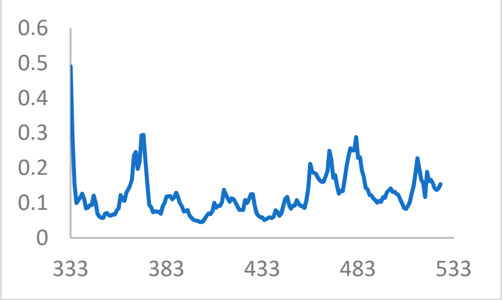

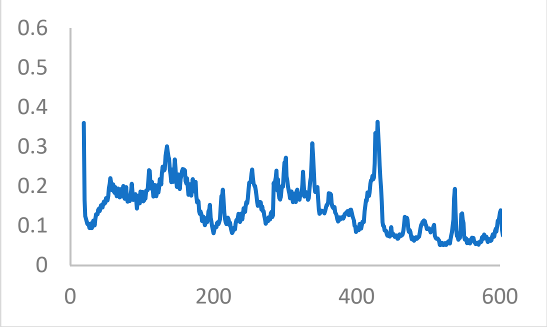

2muts4564

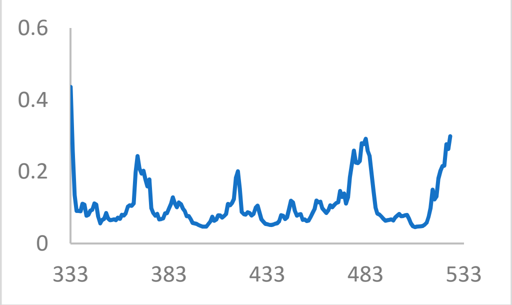

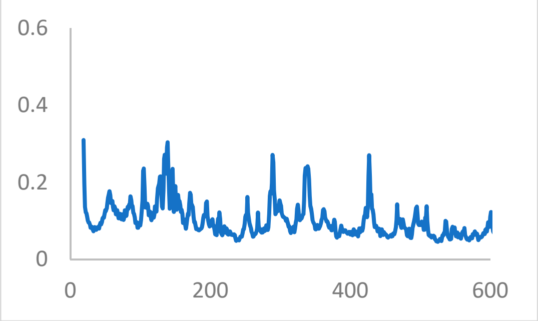

2muts4584

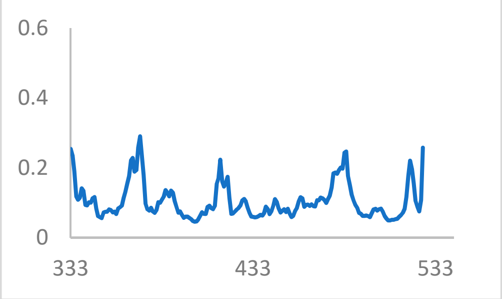

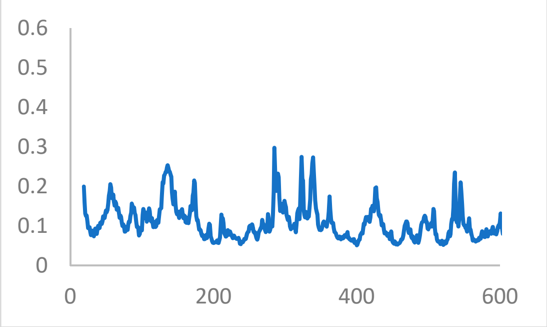

2muts4617

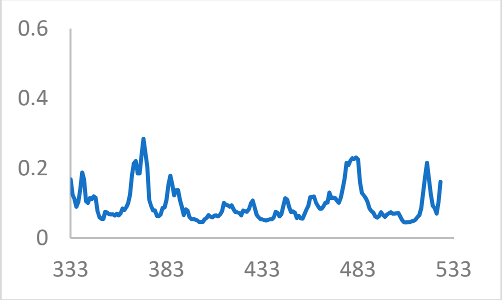

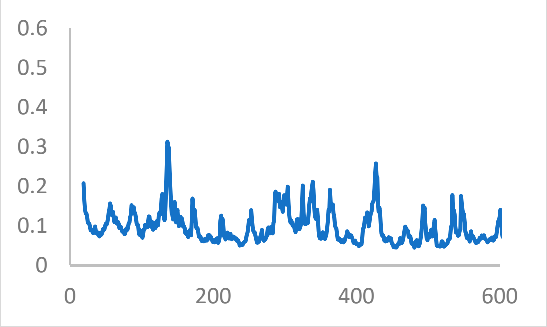

2muts4627

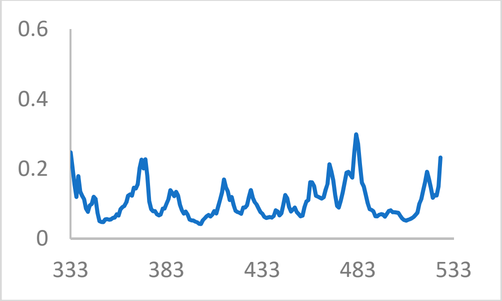

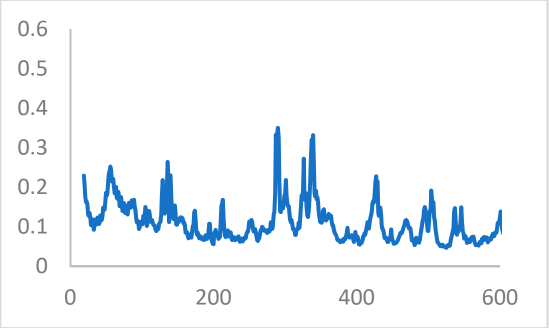

2muts4667

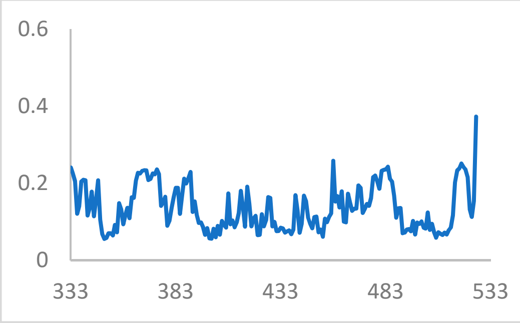

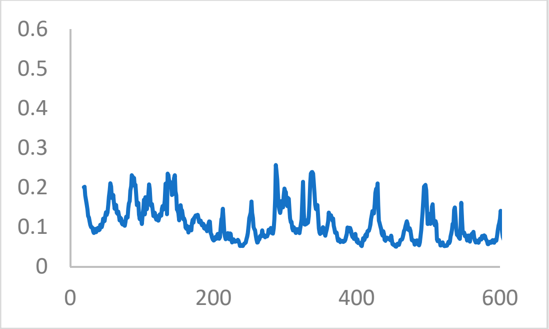

2muts4697

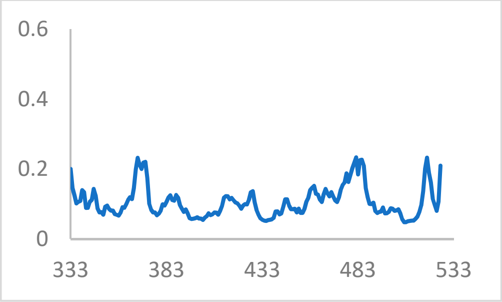

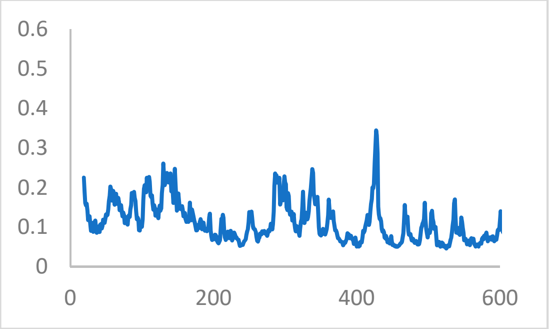

2muts4698

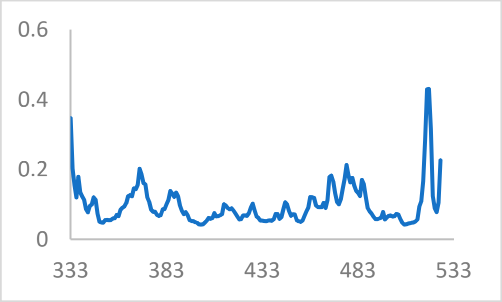

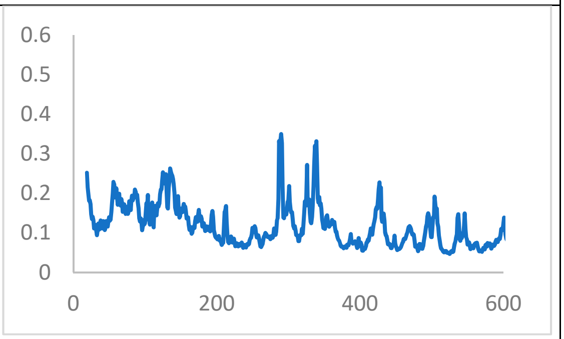

2muts4699

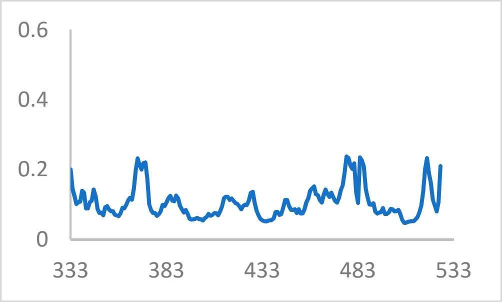

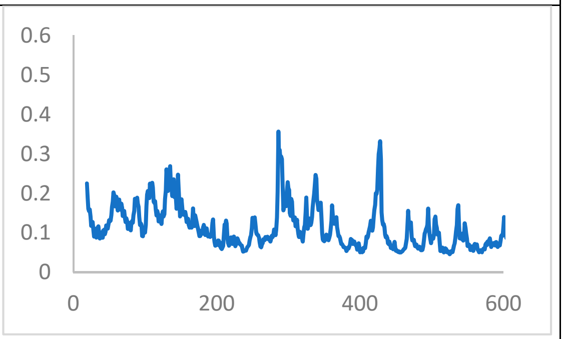

2muts4728	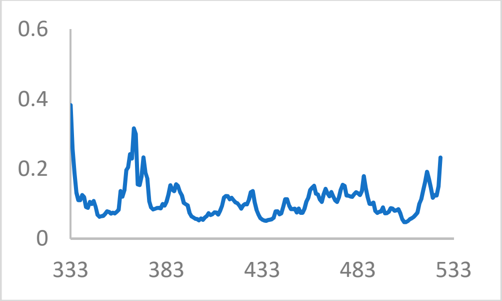	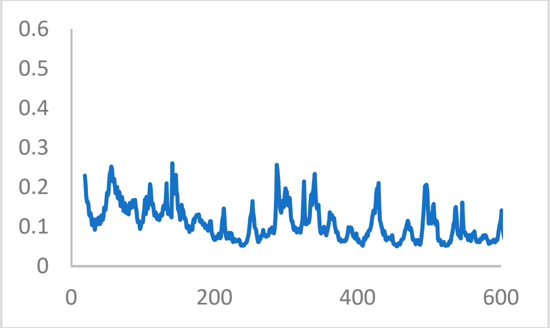
2muts5460	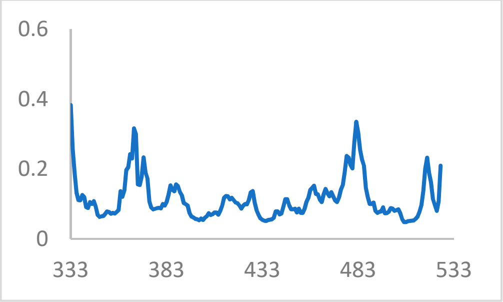	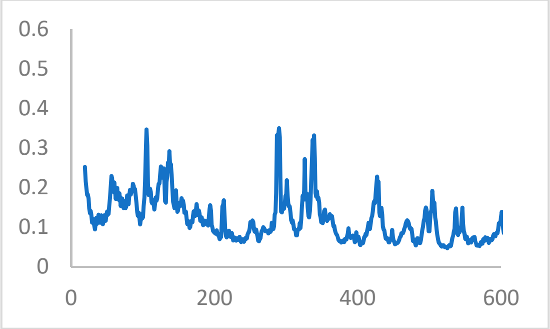
2muts5602	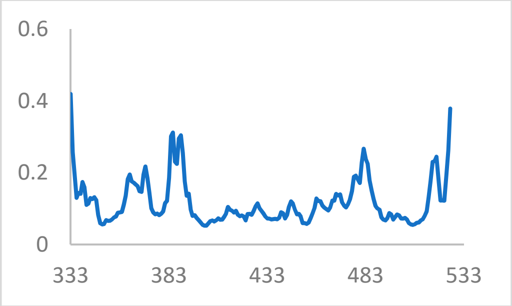	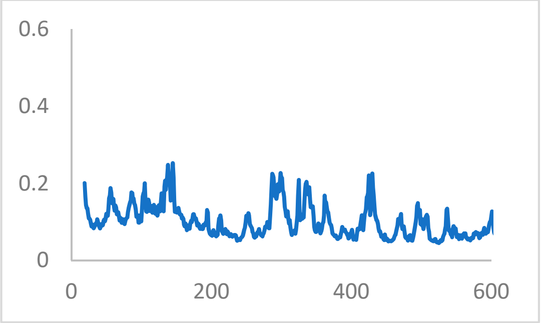
2muts5837	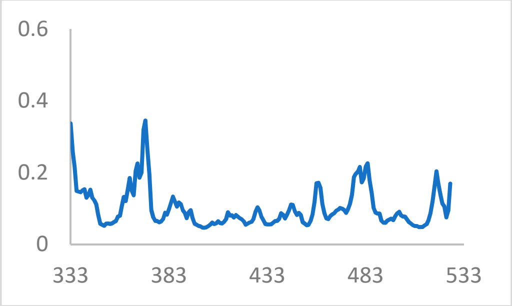	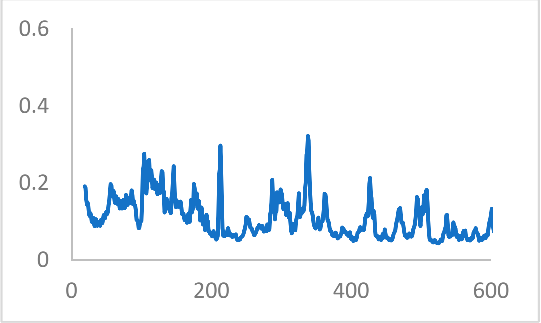
2muts5880	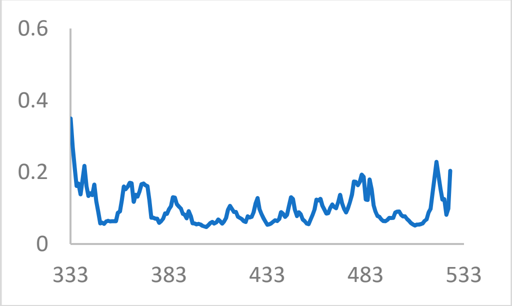	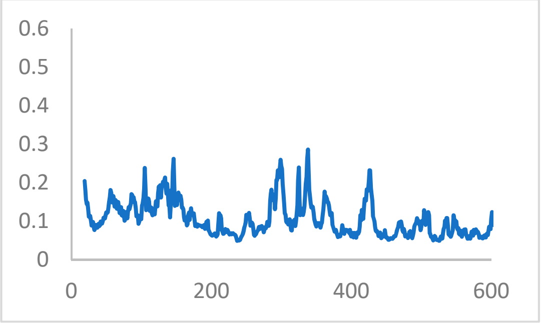

2muts5887	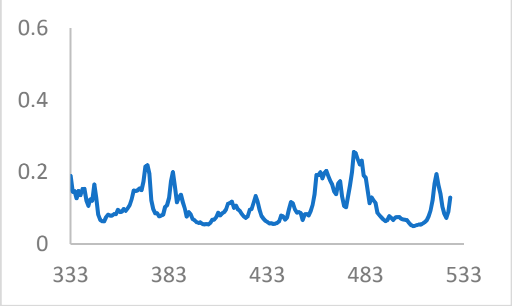	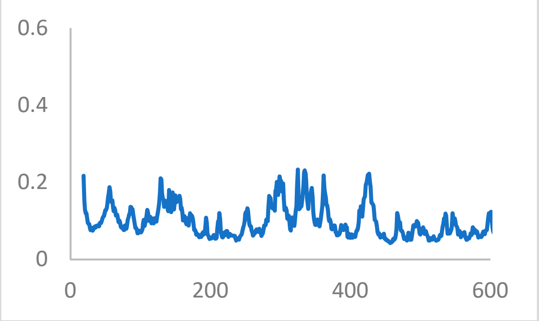
2muts6169	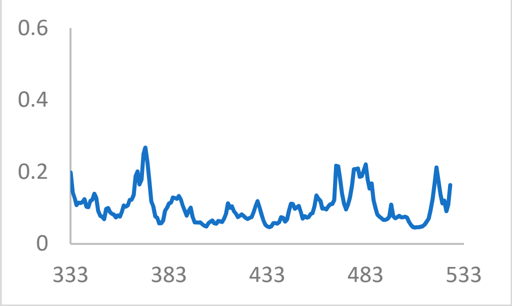	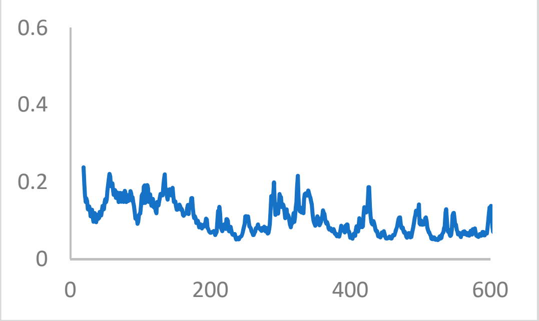
2muts6191	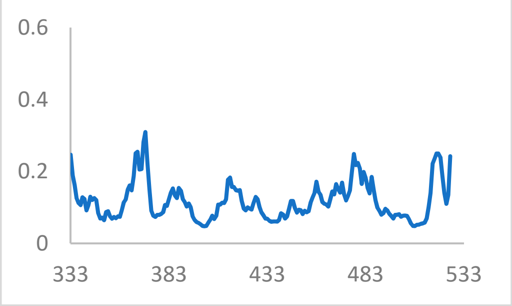	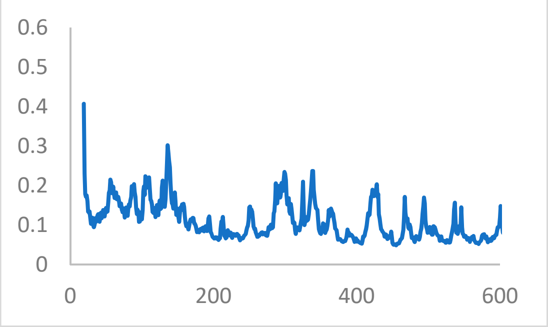
2muts6253	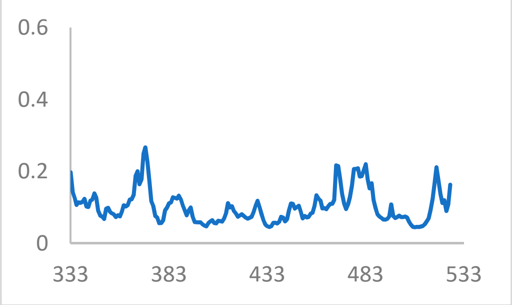	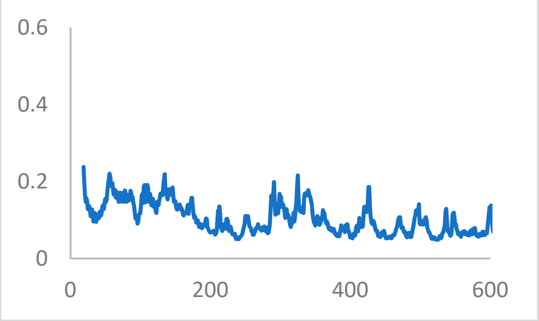
2muts6256	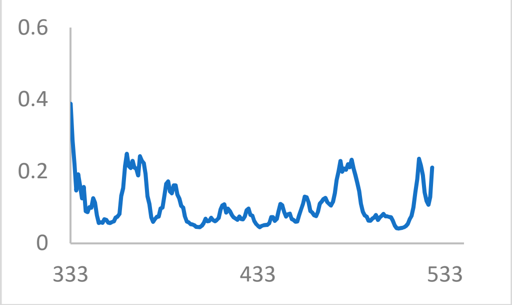	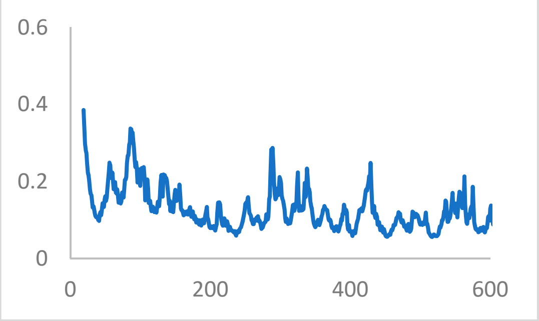

2muts6321	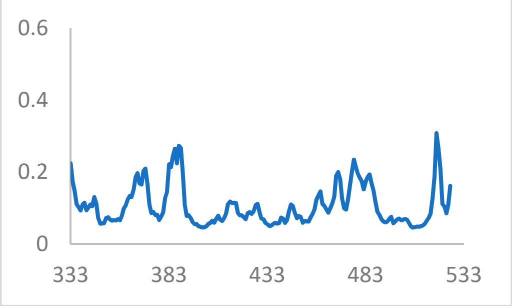	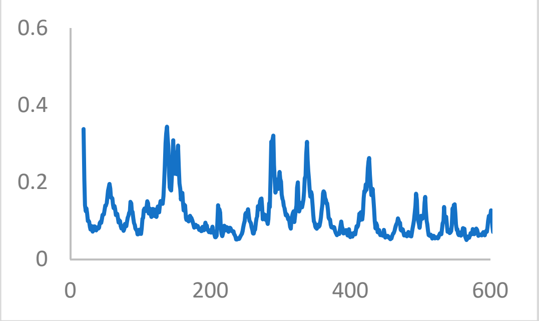
2muts6355	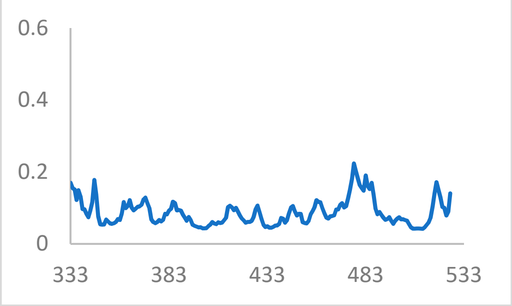	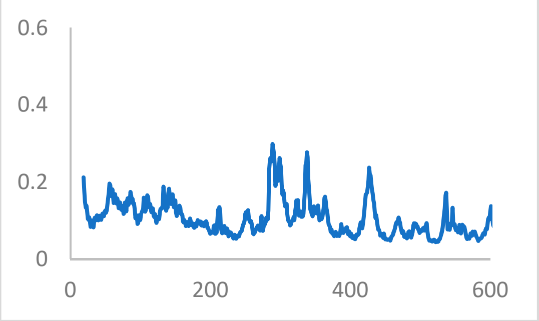
2muts6536	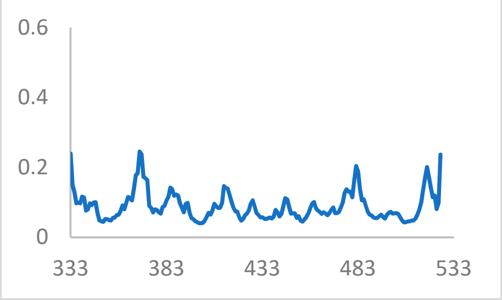	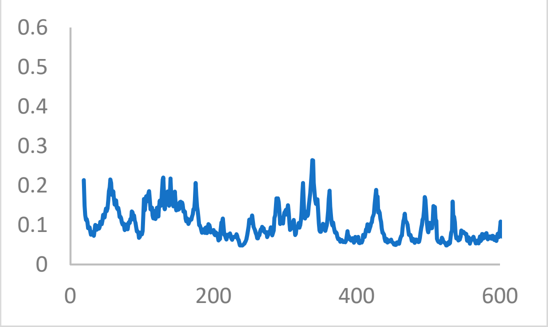
2muts6574	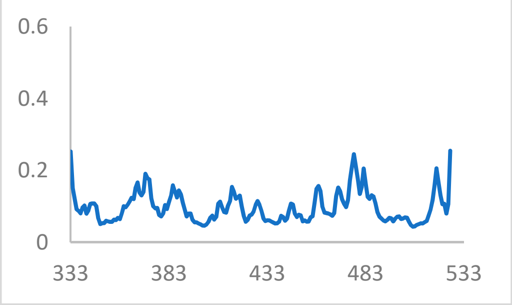	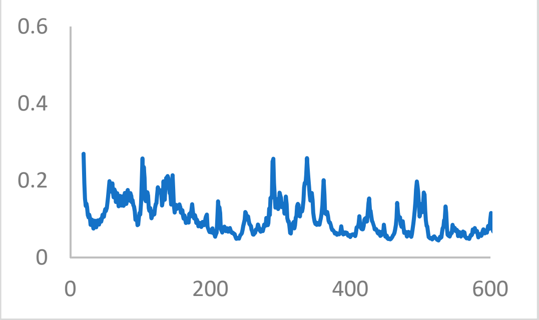
2muts6776	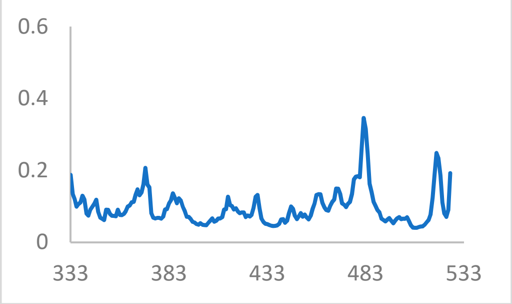	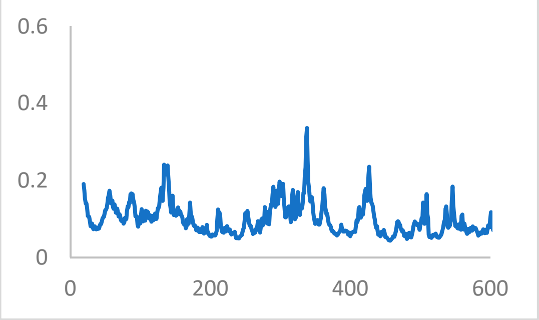

2muts6853

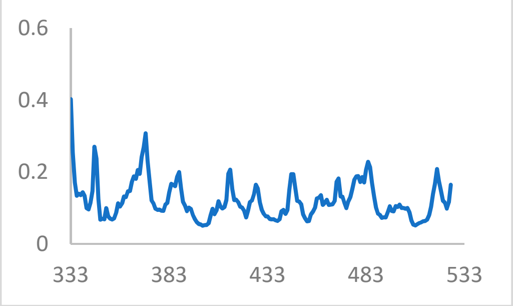

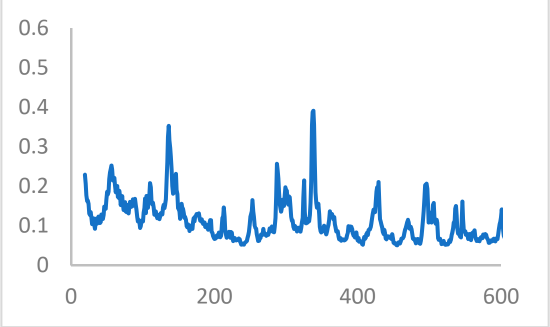

2muts7162

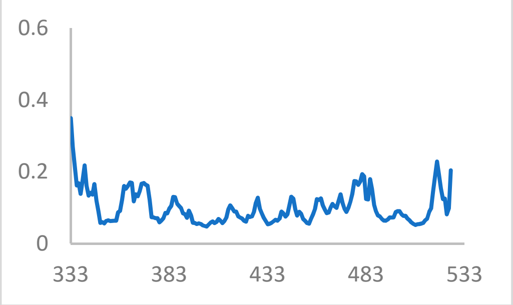

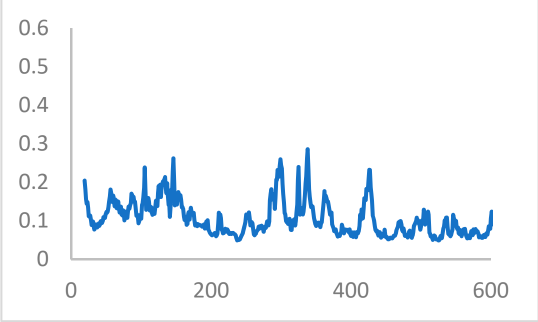

2muts7235

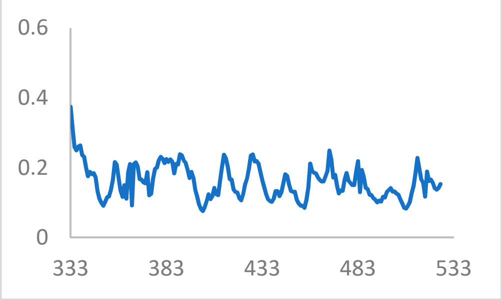

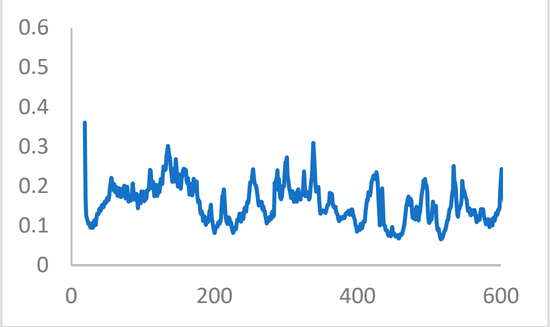

2muts7246

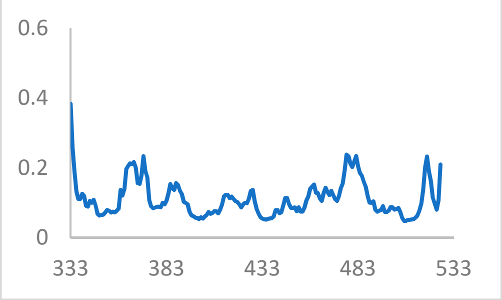

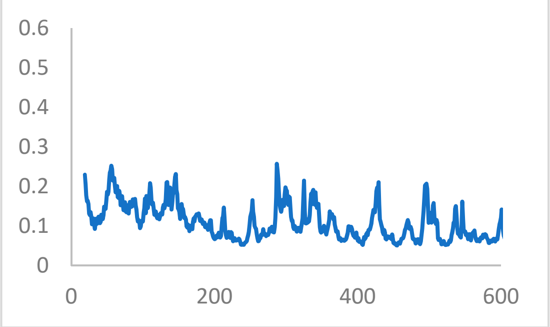

2muts7256

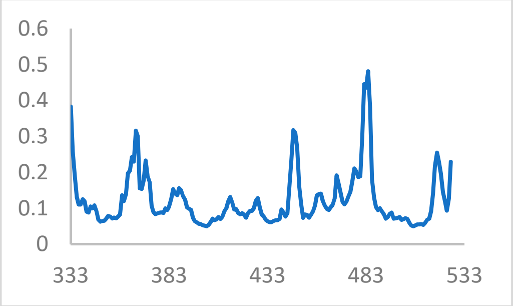

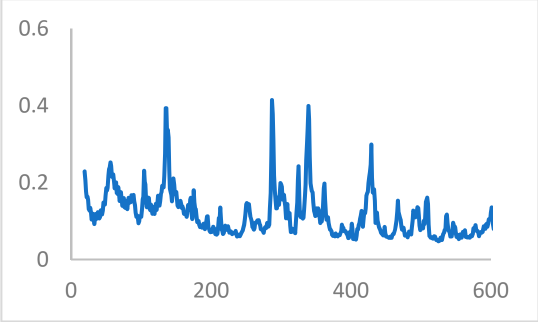

2muts7358	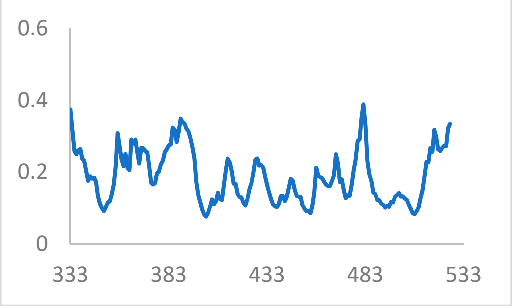	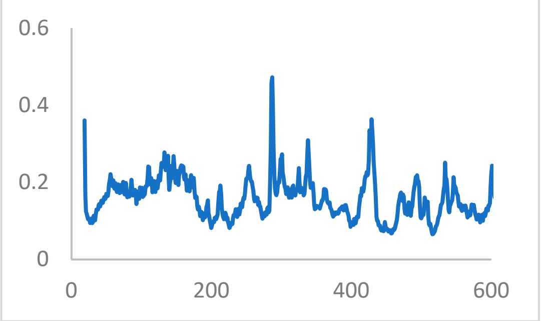
2muts7452	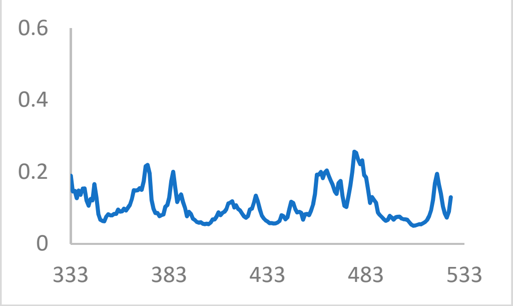	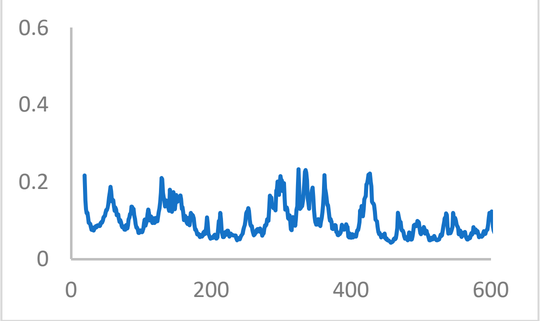
2muts7512	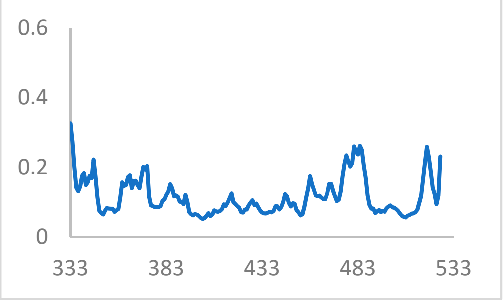	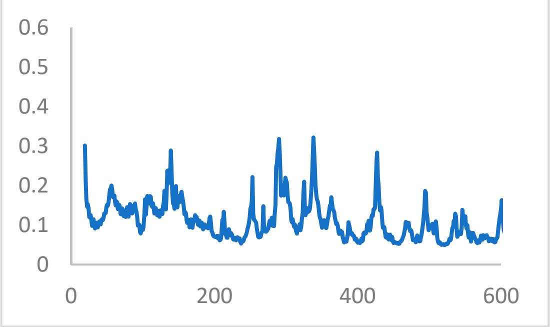
2muts7619	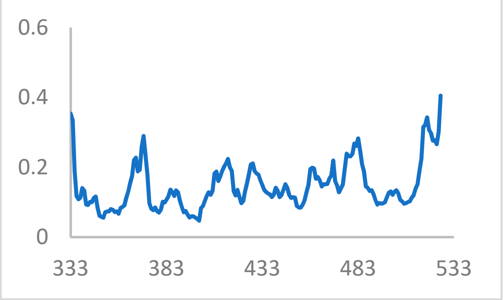	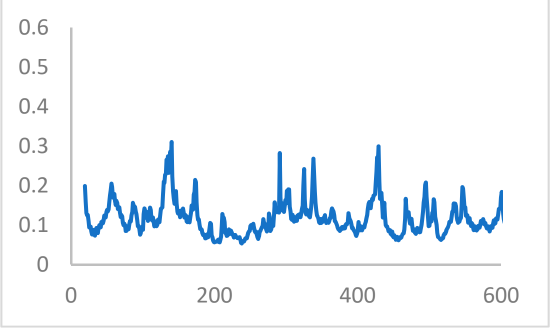
2muts7671	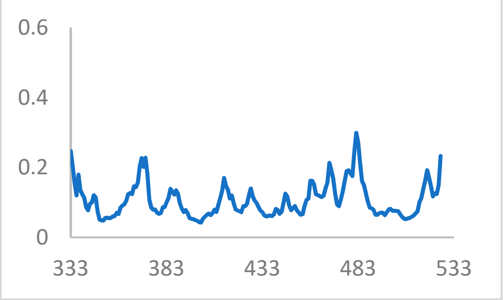	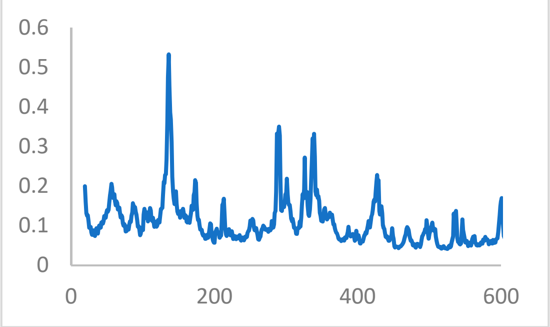

2muts7672

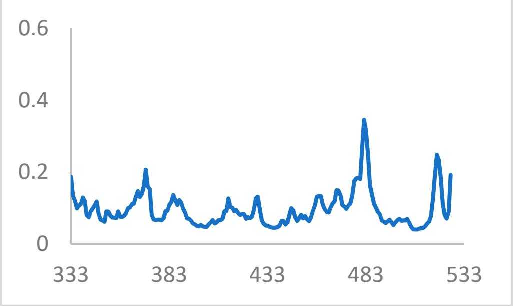

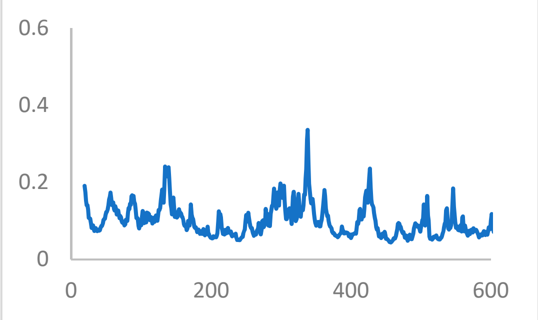

2muts7683

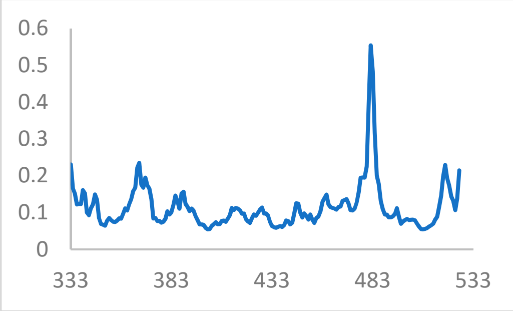

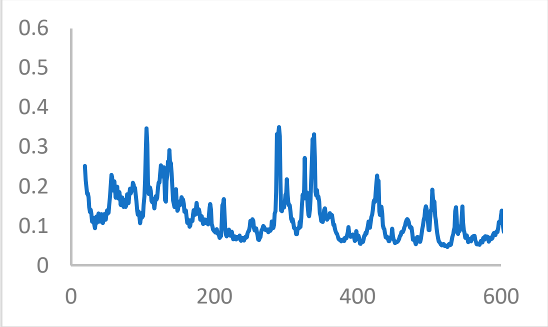

2muts7688

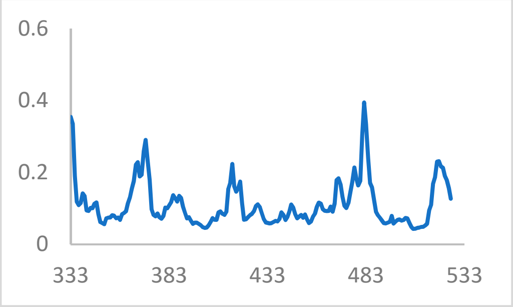

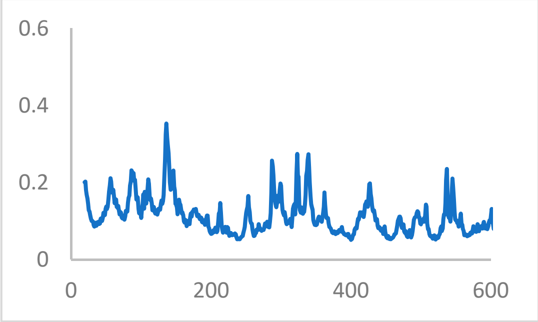

2muts7728

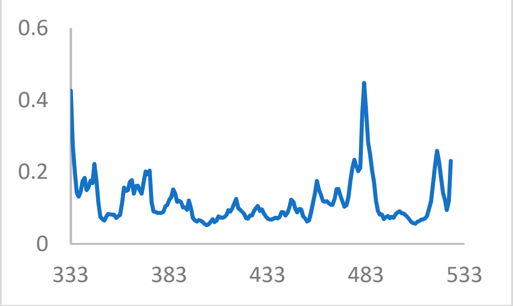

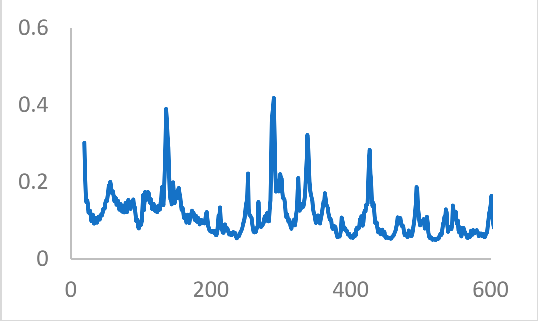

2muts7732

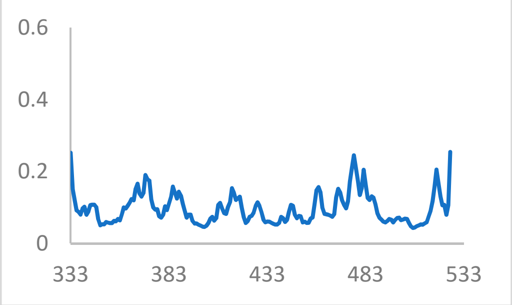

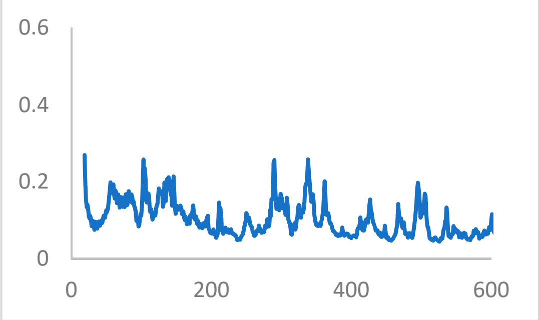

<table>
<tr><td>2muts7736</td><td>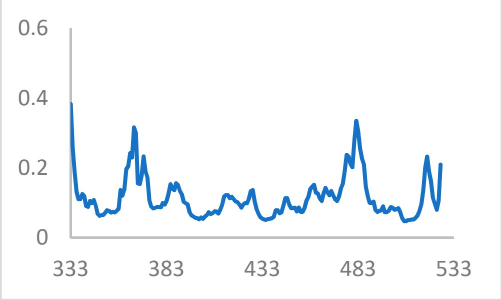
</td><td>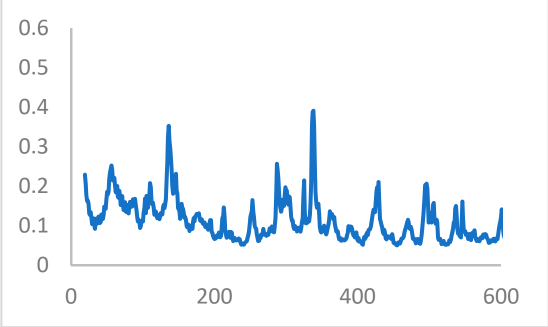
</td></tr>
<tr><td>2muts7850</td><td>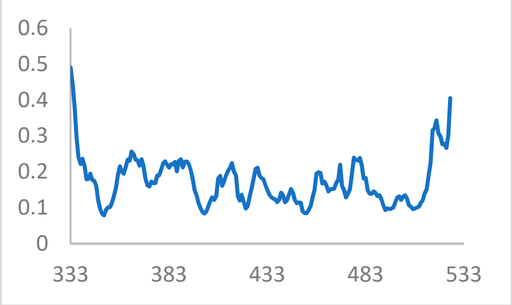
</td><td>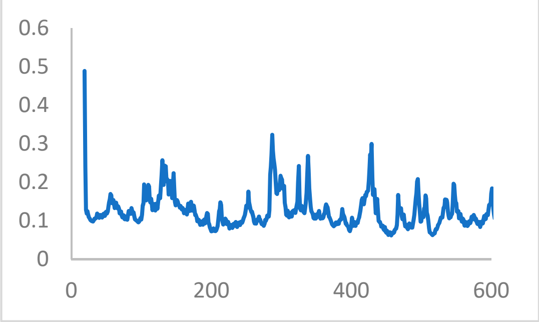
</td></tr>
<tr><td>2muts7889</td><td>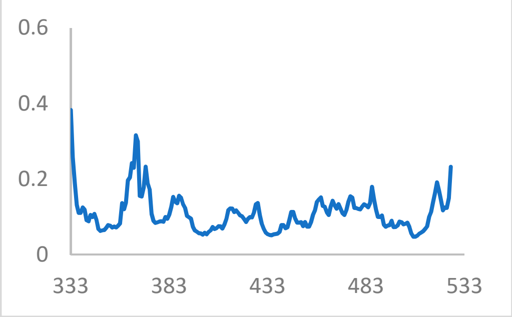
</td><td>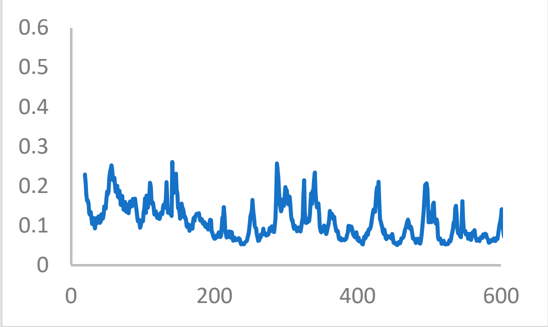
</td></tr>
<tr><td>2muts7941</td><td>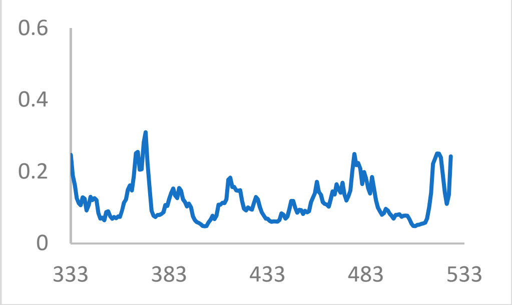
</td><td>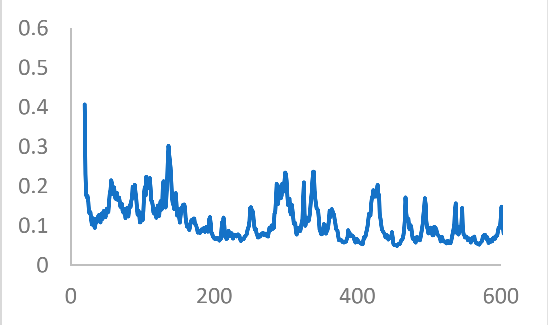
</td></tr>
<tr><td>2muts7999</td><td>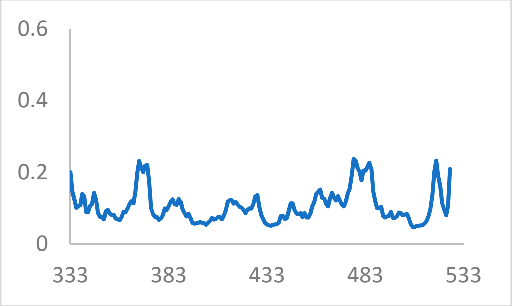
</td><td>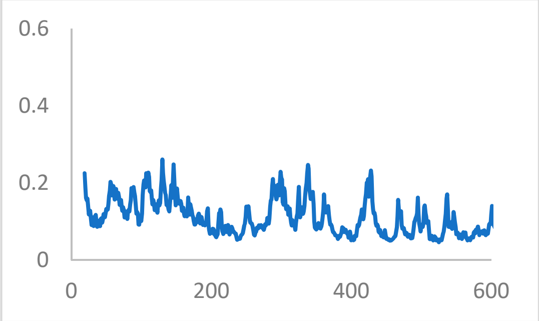
</td></tr>
</table>

2muts8020	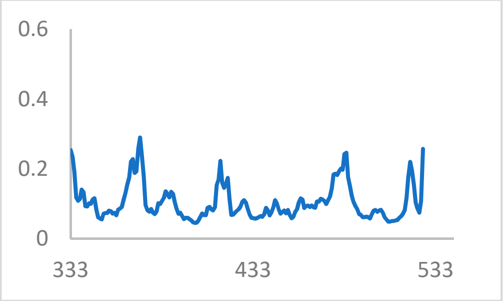	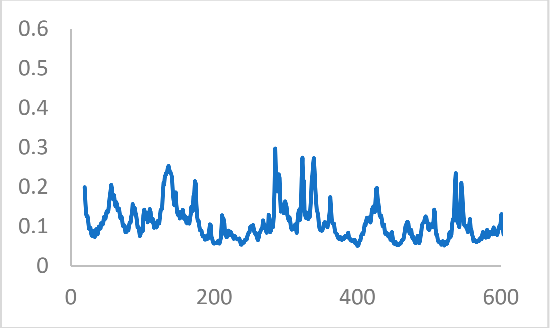
2muts8047	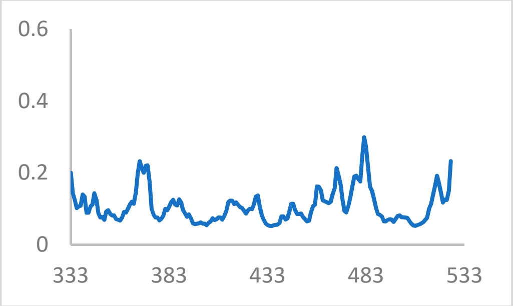	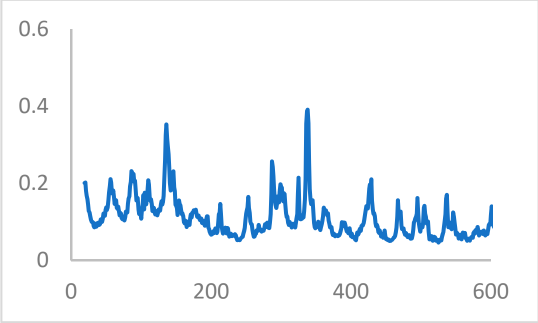
2muts8052	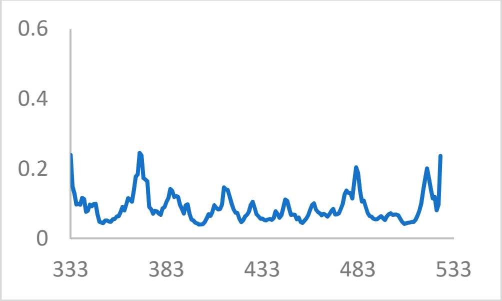	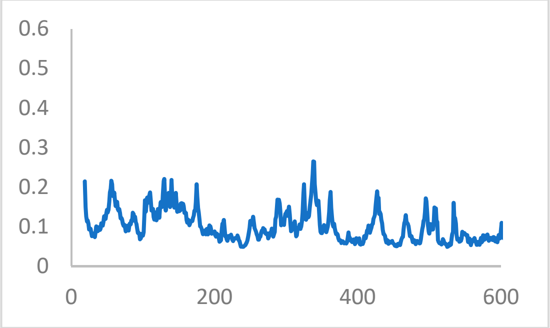
2muts8053	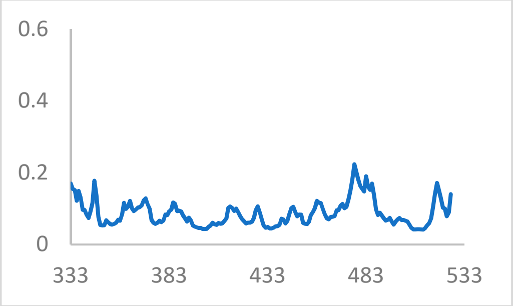	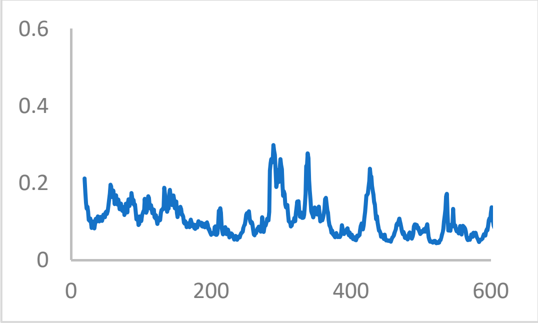
2muts8064	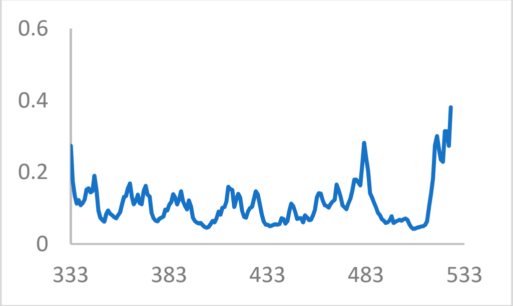	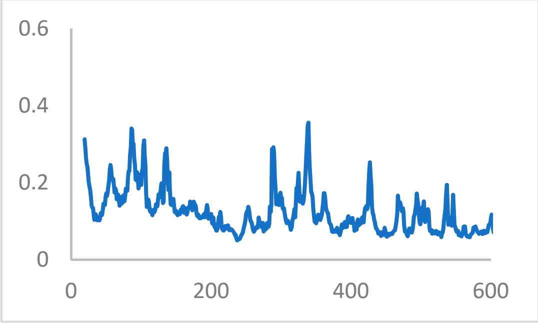

2muts8066	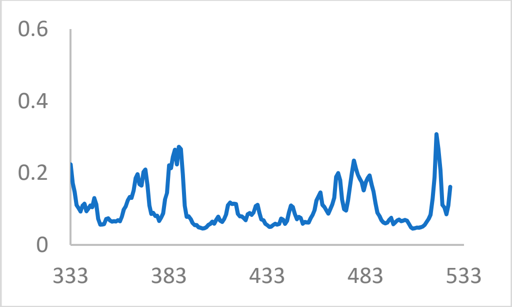	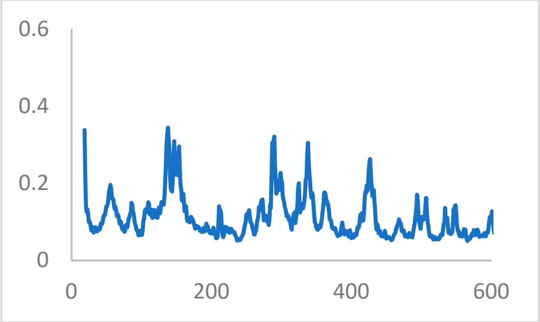
2muts8098	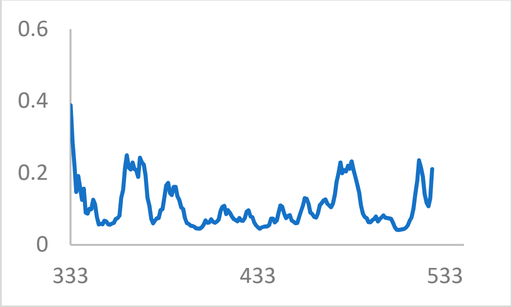	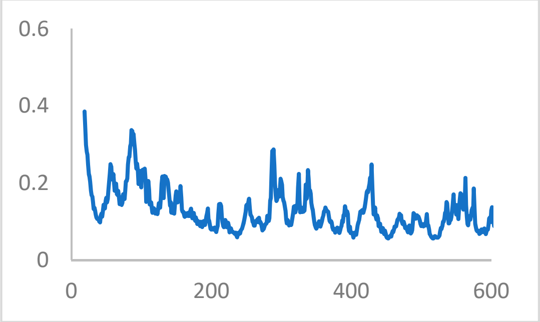
2muts8176	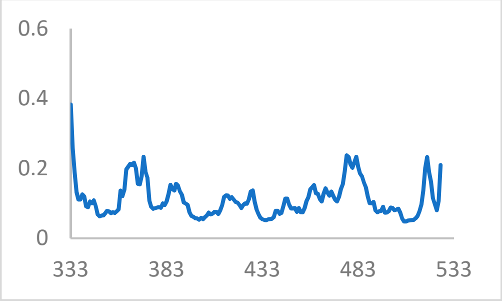	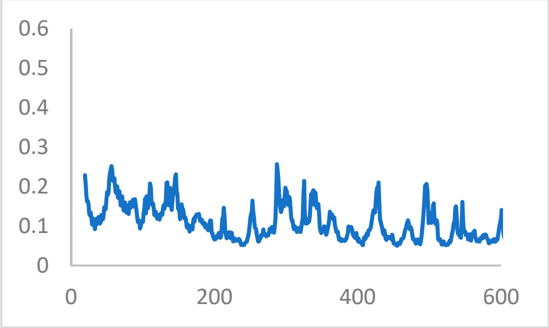
